# Preliminary Study on the Mechanism of the Influence of Saline Oat Pasture on Muscle Metabolism and Meat Quality of Tibetan Sheep

**DOI:** 10.3390/foods14173044

**Published:** 2025-08-29

**Authors:** Xiaoming Xin, Lijuan Han, Shengzhen Hou, Linsheng Gui, Zhenzhen Yuan, Shengnan Sun, Zhiyou Wang, Baochun Yang, Chao Yang

**Affiliations:** College of Agriculture and Animal Husbandry, Qinghai University, Xining 810016, China; hlj880105@163.com (X.X.); 1987990009@qhu.edu.cn (S.H.); 2017990039@qhu.edu.cn (L.G.); 2017990038@qhu.edu.cn (Z.Y.); 2016980007@qhu.edu.cn (S.S.); 1992990011@qhu.edu.cn (Z.W.); 1989990021@qhu.edu.cn (B.Y.); yangchao@qhu.edu.cn (C.Y.)

**Keywords:** saline land, growing oats, Tibetan sheep, meat quality, metabolomics

## Abstract

This study aimed to examine the effects of oats cultivated in saline and non-saline environments on the meat quality and muscle metabolism of Qinghai Tibetan sheep. First, targeted and untargeted metabolomics were used to examine oat quality and metabolites. Second, sheep muscle quality and metabolites were analyzed. Finally, a combined examination of the quality of the oats and their metabolites, as well as that of the muscles, was compared with saline oats. This study hypothesizes that, compared with non-saline environments, soil salinization can improve the nutritional quality of oats, thereby enhancing the meat quality and muscle metabolism of Qinghai Tibetan sheep. Saline-grown oats were shown to have higher levels of crude protein, crude fat, free amino acids, and simple sugar. The meat quality of the sheep fed on saline oats was higher due to free amino acid and carbohydrate metabolism, resulting in improved texture, color, water-holding capacity, and cooked meat percentage, with lower steaming loss. The findings of this study confirm the hypothesis that salinization improves Tibetan sheep meat quality by optimizing oat composition, providing a reference for agricultural and animal husbandry production in saline areas.

## 1. Introduction

The formation of muscle quality is regulated by feed nutrition. As the core feed for ruminants, forage has metabolomic characteristics (such as amino acids, fatty acids, and secondary metabolites) that enter the animal body through digestion and absorption, directly participating in material synthesis and energy metabolism in muscle cells and ultimately affecting the flavor, tenderness, and nutritional composition of meat [1].

Saline soils represent a specialized habitat in terms of biological resources and biodiversity [2]. Saline–alkaline soils are typically rich in soluble salts, such as Na^+^, Cl^−^, SO_4_^2−^, and exchangeable Na^+^, with a total salt content significantly higher than that of non-saline–alkaline soils. High concentrations of Na^+^ can cause an increase in pH, triggering an imbalance of mineral elements. This further leads to elevated contents of mineral elements such as Na, Cl, Ca, and Mg in saline–alkaline soils while reducing the availability of trace elements such as Fe, Mn, and Zn [3]. Changes in the availability of mineral elements in the soil can affect the optimal growth and functional status of plants. The saline–alkaline land in Qinghai Province constitutes 3.2% of the national saline–alkaline land area. The grass in these regions is suitable for the soil conditions, exhibiting enhanced tolerance to cold, drought, and salinity, and serves as feed for animals such as horses, cattle, and sheep [4]. As the primary forage in the saline–alkaline land of Qinghai Province, oats (*Avena sativa* L.) possess a high relative forage value (RFV). Oats grown in saline–alkaline land are rich in essential nutrients, including fats, proteins, and vitamins. They are renowned for their tolerance to cold, drought, and saline–alkaline conditions and are widely regarded as a viable option for the improvement of saline–alkaline land [5]. Liu et al. [6] studied different oat varieties grown in saline and alkaline soils of the Songnen Plain, observing that these oats had high stem-to-leaf ratios, high RFV values, and good relative feeding values. For this reason, they can serve as pioneer plants for the improvement of saline–alkaline soils. Bai et al. [7] investigated the effects of alkaline stress on oats. They found that under alkaline stimulation, the activities of superoxide dismutase (SOD) and peroxidase (POD) in oats increased, along with an increase in the content of soluble sugars. These responses can enhance the alkaline tolerance of oats. Moreover, Yang et al. [8] found that drought stress (DS) can induce peroxidation and osmotic stress in plants, which in turn respond to drought stress by synthesizing osmoprotectants to regulate osmotic pressure. There is insufficient understanding of the disparities in the quality of oats grown on saline compared with non-saline soils in Qinghai.

Tibetan sheep (*Ovis aries*), one of China’s three major basic sheep breeds, is an endemic species to the Qinghai–Tibet Plateau [9]. These sheep are indigenous to Qinghai Province, where they are reared by farmers and herders. They constitute a fundamental basis of the animal husbandry industry in the province [10]. Liang et al. [11] showed that feeding saline forage to sheep can improve the growth performance and feed-to-weight ratio, serum protein metabolism, immunity, antioxidant capacity, and mineral- and flavor-associated compounds in the meat. Qiu et al. [12] investigated the effect of saline alfalfa (*Medicago sativa* L.) on the meat quality, organ development, and serum biochemical indices of meat goats, finding that saline alfalfa was more palatable than conventional alfalfa, with better color, freshness, and tenderness of the meat, and reduced values of cooking and dripping loss. A study by Moreno et al. [13] showed that the meat of lambs fed on saline oats had higher contents of ash, total saturated fatty acids, and polyunsaturated fatty acids, together with lower n-6:n-3 ratios. Pearce et al. [14] discovered that the carcasses of goats fed with halophytes, such as saltbush (*Atriplex* spp.), can yield a higher proportion of lean meat and a lower amount of fat. Furthermore, this method of feeding can increase vitamin E levels, which helps to maintain the meat’s color. Therefore, it was thought that the high metabolite content of oat grasses growing in salt water could improve the quality of Tibetan lamb by encouraging the sheep to store more nutrients and metabolites in their muscles.

There is currently no research on the impact of oat grasses cultivated in saline–alkaline parts of Qinghai on the quality of Tibetan sheep meat. Furthermore, there is no association between saline oats and meat quality. To clarify the mechanism by which oat cultivation in saline soil affects the quality of Tibetan sheep meat, and to identify the key components in oats that influence this change, we conducted the following experiments. Samples were obtained from both saline and non-saline areas in Qinghai Province to investigate nutrient levels and metabolites in oat plants. After a 120-day feeding period, the longissimus dorsi muscles of Tibetan sheep were collected to analyze their eating quality, nutritional quality, and targeted and untargeted metabolites. The relationships between the differential metabolites of the saline oats and the quality of Tibetan sheep, as well as among the differential metabolites, were also examined. The principal metabolites and metabolic pathways by which saline oats affect the quality of Tibetan sheep were identified. The findings serve as a standard for advancing the animal husbandry ecosystem in the Qinghai region, offering extensive data and technical assistance for integrating specific nutrients into standardized feeding protocols.

## 2. Materials and Methods

The Committee of Experimental Animal Care approved all experimental procedures involving animals, while the Qinghai University of Animal Care approved the handling techniques (QUA-2022-0515).

### 2.1. Samples Collection

#### 2.1.1. Oat Sample Collection

Following the standards set by the National Forage Testing Association (NFTA), oats were collected from the saline–alkaline land of Gonghe County, Hainan Prefecture, Qinghai Province, China (GX; latitude: 36°28′2″ N; longitude: 99°16′26″ E; and altitude: 3168.1 m), and non-saline land of Haiyan County, Haibei Prefecture, Qinghai Province, China (YX; latitude: 36°59′36″ N; longitude: 100°55′5″ E; and altitude: 3111 m), and were harvested and preserved at −80 °C for subsequent analysis of oat quality across the distinct regions. The soil sampling areas were consistent with the oat-growing areas, and soil samples were collected in the oat-growing fields of the two locations.

#### 2.1.2. Experimental Design of Oat Feeding in Different Areas of Cultivation

Sixty healthy male sheep, 2 months old and with similar body conditions, were selected and randomly allocated to the Gonghe-housed group (YB, *n* = 30) and the Hai-yan-housed group (B0, *n* = 30). Group YB sheep were raised at Xiangka Meiduo farm in Gonghe County, Hainan Prefecture, Qinghai Province, China, and were fed local saline-cultivated oats. Group B0 sheep were raised at Jinzang Farm in Haiyan County, Haibei Prefecture, Qinghai Province, China, and were fed local non-saline-cultivated oats. Referring to the literature with minor modifications [15,16], both groups were provided with identical feed concentrates and intake, as detailed in Table 1. The two groups of animals (30 in each group) were housed in enclosures with wind-sheltered exercise areas that were also sunny, dry, and well ventilated. The animals were fed twice daily at 08:30 am and 4:30 pm with unrestricted access to feed and water; any feed remaining from the previous feeding time was collected and weighed before the next feeding. Furthermore, the housing was swept, gutters were cleaned daily, and the housing and exercise yards underwent weekly disinfection and sterilization. The fences were maintained in a clean and hygienic condition. All Tibetan sheep underwent immunization, and the transmission of internal and external parasites was systematically prevented and managed. The official 120-day experiment was conducted after a 7-day adaptation period, and slaughtering was initiated at 6 months of age.

#### 2.1.3. Tibetan Sheep Meat Sample Collection

At the end of the feeding trial, six experimental animals were randomly selected from each group and transported to a nearby commercial abattoir. The animals were fasted for 12 h (no food or liquid) and were humanely slaughtered according to animal welfare procedures; i.e., the lambs were stunned and bled. After slaughtering, the Longissimus dorsi lumborum was also removed from one side of each carcass. Slaughtering and sampling were performed together by professionals following uniform standards. All samples were placed in dry ice and transferred to the laboratory for storage at −80 °C for subsequent analysis. Six replicates per group were used for all meat and metabolomics analyses.

### 2.2. Determination of Soil Mineral Elements

Following the method described by Song et al. [17], the concentrations of mineral elements in soil samples were determined using inductively coupled plasma optical emission spectrometry (ICP-OES, Optima 8300, Perkin Elmer, Waltham, MA, USA).

### 2.3. Oat Quality Analysis

#### 2.3.1. Nutritional Values Analysis of Oats

The oats were dried in a blast-drying oven, pulverized, and passed through a 40-mesh sieve to determine their value. Then, according to the method described by Han et al. [18], the moisture content was determined using the oven method (DHG-9070A, Shanghai Bluepard Instruments Co., Shanghai, China), the crude protein content was determined using the Kjeldahl method (K9840, Hanon Advanced Technology Group Co., Jinan, Shandong, China), and the crude fat content was determined using the Soxhlet extraction method (SOx406, Shandong Haineng Scientific Instrument Co., Jinan, Shandong, China). Near-infrared (NIR) spectroscopy (INFRAMATIC 8620) was used to determine acid detergent fiber (ADF) and neutral detergent fiber (NDF) [19]. The acid–base fractionated hydrolysis method was used to determine the crude fiber [20].

#### 2.3.2. Determination of Oats Quality Indices

The relative feeding value (RFV) was calculated as described by Gao [21] using the following formula:

(1) RFV = DMI (%DW) × DDM (%DW)/1.29

where DMI represents the dry matter intake, and DDM indicates the digestible dry matter;

(2) DMI=120/NDF

where NDF indicates the neutral detergent fiber;

(3) DDM=88.9−0.779 × ADF

where ADF represents the acidic detergent fiber.

#### 2.3.3. Free Amino Acid-Targeted Metabolomics Determination of Oats

The samples were extracted from storage at −80 °C and accurately weighed to 60 mg utilizing an electronic balance (AL104, Mettler Toledo, Greifensee, Zurich, Switzerland). Then, 50 µL of water homogenate was incorporated into each sample, followed by vortexing for 60 s with a vortex mixer (QT-1, Shanghai Kit Analytical Instrument Co., Shanghai, China). A solution of methanol (≥99.0%, Fisher Chemical, Pittsburgh, PA, USA) and acetonitrile (≥99.0%, Fisher Chemical, Pittsburgh, PA, USA) (1:1, *v*/*v*) was then introduced in a volume of 400 µL, along with 50 µL of a 50 µM internal standard mixture containing 16 isotopes. The samples were incubated at −20 °C for one hour to precipitate proteins after the mixture was vortexed for 60 s and then subjected to low-temperature sonication using an ultrasonic instrument (JP-100, Shenzhen Jiemeng Cleaning Equipment Co., Shenzhen, Guangdong, China) for two 30 min intervals. Centrifugation was performed at 14,000 rcf and 4 °C for 20 min using a centrifuge (5430R, Eppendorf, Hamburg, Germany). The resulting supernatant was freeze-dried using a vacuum freeze dryer (FD-IC-50, Shanghai Bilang Instrument Co., Shanghai, China) and stored at −80 °C.

Chromatographic separation was conducted using a UHPLC system (1290 Infinity, Agilent, Santa Clara, CA, USA). Standards (≥99.0%, Sigma-Aldrich, St. Louis, MO, USA) were maintained in an autosampler at 4 °C, with the column temperature set to 35 °C. Mass spectrometry analyses were conducted using a mass spectrometer (6500/5500 QTRAP, SCIEX, Framingham, MA, USA) operating in positive ion mode. Quality control (QC) samples were produced by combining aliquots from all samples to evaluate data stability and reproducibility. The relative standard deviation (RSD) for the analyte in the QC samples was under 10%, signifying that the results were stable and reliable.

The distribution diagram of the RSD of free amino acids in the QC samples is presented in the Supplementary Materials, Figure S1A. Table S1 and Formula (S1) of the Supplementary Materials present the relevant standard curves and formulas.

#### 2.3.4. Fatty Acid-Targeted Metabolomics Determination of Oats

The quantitative method was strictly validated following the relevant standards of the International Organization for Standardization (ISO), including validation items such as relative standard deviation (RSD), limit of detection, limit of quantification, and linear range. Detailed data on the method performance characteristics are provided in the Supplementary Materials.

Following the gradual thawing of the sample at 4 °C, 60 mg of the sample was accurately weighed using an electronic analytical balance (AL104, Mettler Toledo, Greifensee, Zurich, Switzerland) and combined with 5 mL of dichloromethane (≥99.0%, Sigma, St. Louis, MO, USA)–methanol (≥99.0%, Fisher Chemical, Pittsburgh, PA, USA) solution (2:1 *v*/*v*). The mixture was thoroughly vortexed, and 2 mL of ultrapure water was added to wash it. The lower phase of the solution was then isolated and evaporated to dryness using a nitrogen stream. Following this, 2 mL of n-hexane was introduced, along with the internal standard, and the mixture underwent methyl esterification for 30 min. Following methylation, 2 mL of ultrapure water was introduced, and 2000 μL of the supernatant was aspirated and evaporated under nitrogen.

The residue was re-dissolved in n-hexane, and the supernatant was transferred into an injection vial for gas chromatography–mass spectrometry (GC-MS) analysis (Agilent, Santa Clara, CA, USA). The samples were separated on a capillary column (19091S-433UI: HP-5ms, 30 m × 250 μm × 0.25 μm, Agilent, America) using a gas chromatography system, with helium as the carrier gas at a flow rate of 1.0 mL/min. Mass spectrometric analysis was conducted using a triple quadrupole mass spectrometer (5977B MSD, Agilent, Santa Clara, CA, USA), and the detection mode was selected ion monitoring (SIM). The QC samples were produced by combining aliquots from all samples to evaluate data stability and reproducibility. The relative standard deviation (RSD) of the analyte in the QC samples was below 10%, signifying reliable and stable results. The internal standard method was used for quantitative analysis, with methyl nonadecanoate (≥99%, NU-CHEK Prep, Inc., Elysian, MN, USA) as the reference material. The calibration process adopted matrix-matched calibration; that is, a series of concentration standard solutions was prepared using blank sample matrices. The linear correlation coefficients (R^2^) of the plotted calibration curves were all greater than 0.99, meeting the requirements of quantitative analysis.

The distribution diagram of the RSD of fatty acids in the QC samples is presented in the Supplementary Materials, Figure S1B. Table S1 and Formula (S2) of the Supplementary Materials present the relevant standard curves and formulas.

#### 2.3.5. Monosaccharide-Targeted Metabolomics Determination of Oats

In this experiment, detection was performed using GC-MS (8890-5977B, Agilent, Santa Clara, CA, USA) with a triple quadrupole mass spectrometer, and the detection mode was selected ion monitoring (SIM). The quantitative method was validated following the relevant standards of the International Organization for Standardization (ISO), including validation items such as relative standard deviation (RSD), limit of detection, limit of quantification, and linear range. Monosaccharide standards were used as calibration standards, and a series of concentration standard solutions was prepared using matrix-matched calibration. The correlation coefficient (R^2^) of the calibration curve was greater than 0.99.

The samples underwent vacuum freeze-drying using a vacuum freeze dryer (CentriVap LABCONCO, Kansas City, MO, USA). They were ground into a powder using a ball mill (MM400, Retsch, Haan, North Rhine-Westphalia, Germany) operating at 30 Hz for 1.5 min. In total, 20 mg of the resultant powder was then measured into appropriately labeled centrifuge tubes. A solvent mixture consisting of methanol (chromatographically pure, Merck, Darmstadt, Hesse, Germany), isopropanol (Merck, Kenilworth, NJ, USA), and water in a volumetric ratio of 3:3:2 (*v*/*v*/*v*) was prepared, and 500 μL of this extract was added to each sample. The samples were vortexed for 3 min and sonicated at 4 °C for 30 min using a multi-tube vortex mixer (MIX-200, Shanghai Jingmei, Shanghai, China). Following this, centrifugation was performed at 4 °C and 12,000 rpm for 3 min using a centrifuge (5424R, Eppendorf, Hamburg, Germany). In total, 50 μL of the supernatant was aspirated, to which 20 μL of an internal standard solution at a concentration of 1000 μg/mL was added. The mixture was subjected to nitrogen evaporation and lyophilization. Subsequently, 100 μL of pyridine methoxide ammonium salt (99%, Sigma-Aldrich, St. Louis, MO, USA) (15 mg/mL) was added, and the samples were incubated at 37 °C for 2 h. Following this, 100 μL of BSTFA (99%, Shanghai Aladdin Biochemical Technology Co., Shanghai, China) was added, and the incubation continued at 37 °C for another 30 min (Thermo Scientific Forma 311, Thermo Fisher Scientific, Waltham, MA, USA).

Over 80% of the compounds in the QC samples had coefficient of variation (CV) values below 0.3, signifying the stability of the experimental data. Moreover, the proportion of compounds exhibiting CV values below 0.2 in the QC samples surpassed 80%, indicating substantial data stability.

Table S2 and Formula (S3) of the Supplementary Materials present the relevant standard curves and formulas. The parameters of GC-MS are shown in the Supplementary Materials, Table S3.

#### 2.3.6. Untargeted Metabolomics Determination of Oats

After the samples were slowly thawed at 4 °C, 60 mg of each sample was weighed using an electronic balance and added to pre-chilled methanol (≥99.0%, Fisher Chemical, Pittsburgh, PA, USA)–acetonitrile (≥99.0%, Fisher Chemical, Pittsburgh, PA, USA)–water solution (2:2:1, *v*/*v*). The mixture was homogenized using vortexing with a vortex mixer (QT-1, Shanghai Kit Analytical Instrument Co., Shanghai, China) and then subjected to low-temperature sonication for 30 min using an ultrasonic cleaner (KQ5200E, Kunshan Shumei, Kunshan, Jiangsu, China). Subsequently, the samples were incubated at −20 °C for 10 min. After that, the samples were centrifuged at 14,000 rpm for 20 min at 4 °C using a centrifuge (5430R, Eppendorf, Hamburg, Germany). The supernatant was collected and dried under a vacuum (FD-IC-50, Shanghai Bilang Instrument Co., Shanghai, China). For mass spectrometry analysis, the dried residue was reconstituted in 100 μL of an acetonitrile–water solution (1:1, *v*/*v*), vortexed again, and then centrifuged at 14,000× *g* for 15 min at 4 °C. Finally, the supernatant was injected for analysis.

The samples were separated using a UHPLC system (1290 Infinity LC, Agilent, Santa Clara, CA, USA) with a HILIC column (ACQUITY UPLC BEH Amide 1.7 μm, 2.1 mm× 100 mm column, Waters, Milford, MA, USA). The column temperature was maintained at 25 °C, the flow rate was set at 0.5 mL/min, and the injection volume was 2 μL. The mobile phase consisted of two components: mobile phase A consisted of a mixture of water, 25 mM ammonium acetate (≥99.0%, Sigma-Aldrich, St. Louis, MO, USA), and 25 mM Ammonia solution, while mobile phase B consisted of acetonitrile (≥99.0%, Fisher Chemical, Pittsburgh, PA, USA). The gradient elution schedule was 0.5–7 min, and the fraction of mobile phase B linearly reduced from 95% to 65%. From 7 to 8 min, it declined linearly from 65% to 40%. From 8 to 9 min, the proportion of mobile phase B remained constant at 40%. From 9 to 9.1 min, the proportion of mobile phase B produced linearly from 40% to 95%. From 9.1 to 12 min, the proportion of mobile phase B was sustained at 95%. The samples were maintained in an autosampler at 4 °C during the complete analysis process.

Mass spectrometry analysis was conducted using Q Exactive-series mass spectrometers (Thermo Fisher Scientific, Waltham, MA, USA), with detection performed in both the positive and negative electrospray ionization (ESI) modes. The parameters for the ESI source and mass spectrometry settings were as follows: nebulizing gas and auxiliary heating gas 1 (Gas1): 60; auxiliary heating gas 2 (Gas2): 60; curtain gas (CUR): 30 psi; ion source temperature: 600 °C; and spray voltage (ISVF): ±5500 V (for both positive and negative modes). The mass spectrometry acquisition mode was full scan. The primary mass-to-charge ratio detection range was 80–1200 Da with a resolution of 60,000 and a scan accumulation time of 100 ms. The secondary level adopted a segmented acquisition method, with a scanning range of 70–1200 Da; a secondary resolution of 30,000; and a scan accumulation time of 50 ms.

Raw data were converted to the mzXML format using ProteoWizard, followed by peak alignment, retention time correction, and peak area extraction via the XCMS software (version 3.14.0, Scripps Research, La Jolla, CA, USA). The extracted data underwent initial metabolite annotation through the combined use of these two tools, with subsequent structural confirmation referencing the Human Metabolome Database (HMDB) and Kyoto Encyclopedia of Genes and Genomes (KEGG). Fragmentation spectra obtained using liquid chromatography–high resolution tandem mass spectrometry (LC-HRMS/MS) were utilized, where MS/MS data assisted in accurate metabolite annotation to ensure the reliability of identification results. The identified metabolites were further subjected to data preprocessing, and their functions and involved metabolic pathways were determined using HMDB and KEGG.

### 2.4. Determination of Meat Quality 

#### 2.4.1. Determination of Carcass Traits

Carcass segmentation involved measuring the thickness of rib meat, abdominal wall, backfat, and the eye muscle area, as outlined by Ma et al. [22].

The area of the eye muscle was determined at the cross-section between the 12th and 13th ribs of the Tibetan sheep during carcass segmentation. This cross-section was outlined using sulfuric acid paper, and then, the area was calculated with a 1 cm × 1 cm grid. The rib thickness was measured as the tissue thickness 110 mm from the 12th and 13th ribs to the midline of the spine in the Tibetan sheep. The thickness of the abdominal wall was assessed at a point 127 mm from the 12th and 13th ribs. Furthermore, the backfat thickness was measured as the fat layer directly above the center of the eye muscle between the 12th and 13th ribs of the Tibetan sheep.

#### 2.4.2. Determination of Meat-Eating Quality 

Qualities associated with eating, including pH, color, thawing loss, cooking loss, cooked meat percentage, and texture, were determined using the method of Zhang et al. [23] with slight modifications.

Briefly, the pH_45min_ and pH_24h_ were determined by inserting a portable pH meter (PHS-3C, Shanghai Leici Instrument Factory, Shanghai, China) into the meat samples at a depth of 2–3 cm. We calibrated the pH meter using pH 4.0 and 6.86. An automatic colorimeter (ADCI-60-C, Beijing Chentaike Instrument Technology Co., Beijing, China) was used to measure the values of L* (lightness), a* (redness), and b* (yellowness) on the meat surface. The colorimeter was equipped with a standard xenon lamp within the close aperture of 8 mm set to Illuminant D65 with an observer angle of 2°. In addition, the meat samples were heated in a water bath at a constant temperature of 80 °C until the internal temperature reached 70 °C. Next, using an iron ruler and a scalpel, the samples were cut into 3 cm × 1 cm × 1 cm meat columns in the direction of the muscle fibers. A muscle tenderness meter (RH-N50, Guangzhou Runhu Instrument Co., Guangzhou, Guangdong, China) was then used in the direction of the vertical muscle fiber to evaluate the shear force.

The water-holding capacity was calculated as the ratio of the amount of water lost by the meat samples to the initial weight of the samples, which was determined after the meat samples (1 cm × 1 cm × 1 cm) were subjected to a pressure of 350 N by a water-holding capacity tester (RH-1000, Guangzhou Runhu Instrument Co., Guangzhou, Guangdong, China). The cooked meat percentage was calculated as the ratio of the weight of the meat samples after being boiled in a water bath at 80 °C for 40 min to the initial weight of the samples (average weight of 60 g). For cooking loss analysis, the meat samples (2 cm × 3 cm × 2 cm) were cooked in a water bath at 80 °C for 30 min, and cooking loss was calculated as a percentage of the weight change in the samples from the initial weight of the samples. In the same way, the thawing loss was calculated as the ratio of the weight of the meat samples after being unfrozen in a refrigerator at 4 °C for 12 h to the initial weight of the samples (average weight of 30 g). Next, a texture profile analysis (TPA) analyzer (TA.XTC-18, Shanghai Baosheng Industrial Development Co., Shanghai, China) was used to measure the hardness, elasticity, adhesion, cohesion, and chewiness of the samples (1 cm × 1 cm × 1 cm). The probe model utilized was TA3/100, while the fixture model was TA-RT-KIT.

#### 2.4.3. Determination of Meat Sensory Evaluation 

This study references the standards ISO 13299:2016 (en) and ISO 5492 for the sensory evaluation methodology, specifically focusing on establishing a sensory profile for two groups of Tibetan sheep samples.

Approximately 100 g of Tibetan sheep meat samples from each group were weighed, and the two groups were individually labeled. These samples were then cooked in a thermostatically regulated water bath (HH-6, Changzhou Langyue Instrument Manufacturing Co., Nanjing, Jiangsu, China) at a constant temperature of 100 °C. Once the sheep’s internal temperature fell between 60 and 70 °C, the samples were taken from the water bath. The cooked sheep was cut further into equally sized and shaped cubes, each weighing roughly 3 g, guaranteeing the objectivity of the evaluation by the panelists. A sensory evaluation panel of 15 members assessed the color, aroma, juiciness, taste, texture, and general acceptability of the Tibetan sheep samples in each group. The more detailed sensory evaluation is shown in the Supplementary Materials, Table S4.

#### 2.4.4. Determination of Meat Nutritional Quality 

The method of the AOAC (2005) was used to determine the moisture, crude fat, and crude protein contents of the meat samples [24]. In brief, the moisture content of the muscle was determined using direct drying in an oven (DHG-9070A, Shanghai Bluepard Instruments Co., Shanghai, China) at 105 °C until a constant weight was obtained. The crude fat content was assessed using the Soxhlet extraction method (SOx406, Shandong Haineng Scientific Instrument Co., Jinan, Shandong, China). Moreover, the content of crude protein was measured using the Kjeldahl nitrogen method (K9840, Hanon Advanced Technology Group Co., Jinan, Shandong, China).

#### 2.4.5. Free Amino Acid and Fatty Acid Contents in Tibetan Sheep Meat

The free amino acid and fatty acid contents in the Tibetan sheep meat were determined using the methods described in Section 2.3.3 and Section 2.3.4, respectively.

#### 2.4.6. Untargeted Metabolomics Determination of Tibetan Sheep Meat

The untargeted metabolomics analysis of Tibetan sheep meat was performed using the methods described in Section 2.3.6.

### 2.5. Data Processing and Analysis

The data were expressed as mean ± standard deviation (SD) and analyzed using independent samples t-tests in SPSS version 26.0 (IBM Corp., Armonk, NY, USA), with *p* < 0.05 considered statistically significant. Following sum-normalization, the data were processed and then subjected to multivariate data analysis using the R package (ropls) (version 1.26.0, maintained by Etienne Thevenot, Lyon, Auvergne-Rhône-Alpes, France), which included partial least squares discriminant analysis (PLS-DA), orthogonal partial least squares discriminant analysis (OPLS-DA), and Pareto-scaled principal component analysis (PCA). To determine each variable’s contribution to the classification, the variable importance in the projection (VIP) value was computed for each variable in the OPLS-DA model. The associations between meat quality and meat and oat metabolites were assessed using Pearson’s correlation coefficients, with *p* < 0.05 considered statistically significant.

## 3. Results

### 3.1. Analysis of Soil Mineral Elements

As shown in Table 2, both types of soils had relatively high contents of elements such as Al, Ca, Fe, K, Mg, and Na, while the contents of trace elements, including Cu, V, Co, Rb, Se, and Ni, were all less than 100 mg·kg^−1^. Specifically, the contents of Al, Ca, K, Mg, Na, and S in saline–alkaline soils were significantly higher than those in non-saline–alkaline soils, whereas the contents of Fe, P, and Mn were significantly lower (*p* < 0.001). In addition, all trace elements in non-saline–alkaline soils were significantly higher than those in saline–alkaline soils.

### 3.2. Regional Variations in Oat Nutritional Quality and Metabolite Composition

#### 3.2.1. Analysis of Oat Nutritional Values

There were significant differences in the nutritional quality of oats grown in different regions. Table 3 demonstrates that the crude protein levels in the GX group (9.03%) were significantly elevated compared with the YX group (7.16%) (*p* < 0.01). The crude fat content in the YX group was 7.45 g/kg, which is significantly lower than that of the GX group (14.92 g/kg). The neutral detergent fiber content showed a significant difference between the two groups (*p* < 0.01), which was significantly lower in the GX group than in the YX group.

#### 3.2.2. Calculation of Oat Quality Indices

As demonstrated in Table 3, the GX group had higher DMI and RFV values (*p* < 0.01) than the YX group, suggesting that the oats in the GX group provided higher feeding values for ruminants. The digestible dry matter (DDM) and dry matter intake (DMI) values were used to evaluate the quality of the oats grown in various regions. They were negatively correlated with ADF and NDF contents.

#### 3.2.3. Targeted Metabolomics Analysis of Oats

##### Free Amino Acid-Targeted Metabolomics Analysis of Oats

Table 3 shows significant differences in the free amino acid compositions and contents of oats grown in different regions. Overall, oats grown in saline conditions had significantly higher levels of essential free amino acids (EAAs), non-essential free amino acids (NEAAs), and total free amino acids (TAAs) than oats grown in non-saline soils, which implies that saline environments may promote the accumulation of free amino acids in oats. Glycine, serine, and threonine were more abundant in the saline oats. Furthermore, the GX group had higher valine, lysine, arginine, alanine, phenylalanine, and methionine levels than the YX group, indicating significant regional variations.

##### Fatty-Acid-Targeted Metabolomics Analysis of Oats

As shown in Table 3, a variety of common fatty acids were detected in the oats, with palmitic acid showing the highest levels in the saline oats (259.896 ± 19.93). Similarly, there were significant regional differences in the contents of linoleic acid, palmitoleic acid, arachidic acid, and α-linolenic acid: all were higher in saline than in non-saline areas. In the GX group, the contents of PUFA (302.81 ± 13.24) and SFA (55.37 ± 1.22) were significantly higher than those in the YX group. Similarly, the contents of omega-3 (261.11 ± 17.30) and omega-6 (41.71 ± 4.06) in the GX group were also significantly higher than those in the YX group. The two groups had no significant differences in MUFA, EPA, and DHA contents. However, the TFA content in the GX group (365.91 ± 12.09) was significantly higher than in the YX group.

##### Monosaccharide-Targeted Metabolomics Analysis in Oats

As shown in Table 3, 25 differential saccharides were detected in the oats from different regions, with sucrose representing the most abundant sugar from the saline–alkaline areas. Moreover, the levels of glucose, mannose, galactose, arabinose, and maltose also differed significantly between the regions, being higher in oats from saline soils than from non-saline land (*p* < 0.05).

#### 3.2.4. Untargeted Metabolomics Analysis of Oats Metabolites

##### Identification and Analysis of Differential Metabolites in Oats

Figure S2A,B in the Supplementary Materials show that the spectra and the total ion chromatogram (TIC) of the quality control (QC) samples were compared and overlapped. The experimental findings demonstrated slight variance due to instrumental error because the response intensities and retention periods of the chromatographic peaks were identical.

The peaks obtained from the extraction of all experimental and QC samples were analyzed using PCA and are shown in the Supplementary Materials, Figure S3A,B. This suggests that the studies were reproducible because the QC samples were closely clustered in positive and negative ion modes. After the QC samples were eliminated, PLS-DA and OPLS-DA were run to improve group differentiation. The GX and YX groups demonstrated intra-group clustering and inter-group separation in both analysis modes, with the OPLS-DA results demonstrating more pronounced effects (see Supplementary Materials, Figure S3C,D). To prevent the supervised model from being overfitted during the modeling phase, a replacement test was employed to test the model and guarantee its validity. Figure S3E,F in the Supplementary Materials show the replacement test plots of the OPLS-DA model, in which the replacement retention gradually decreased, and both R^2^ and Q^2^ of the stochastic model gradually decreased, indicating that the original model did not suffer from overfitting and that the model showed good stability.

##### Bioinformatics Analysis of Differential Metabolites in Oats

**Figure 1:**
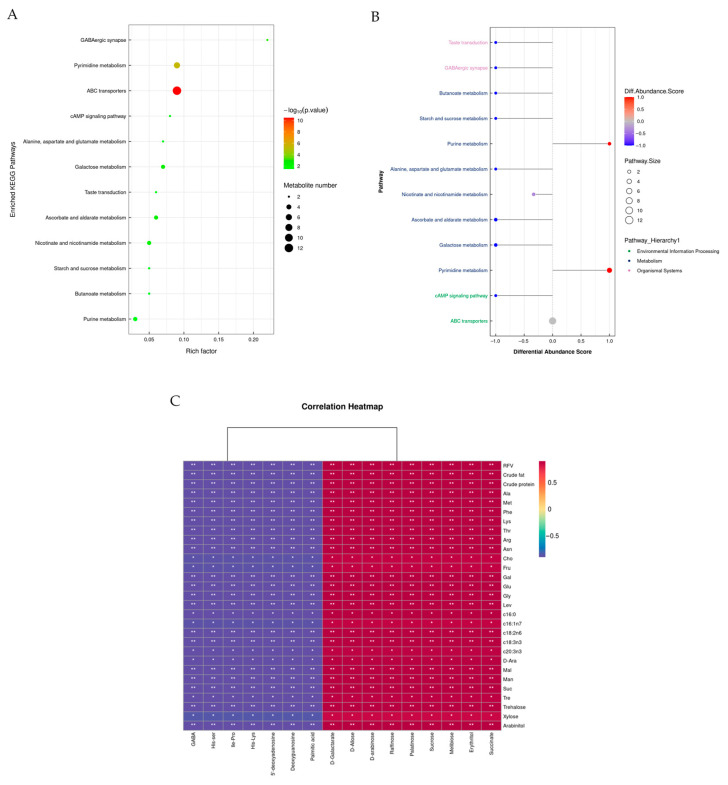
(**A**) Top 12 enriched KEGG pathways of the comparison between YX and GX groups, (**B**) a differential abundance score map of differential metabolic pathways, (**C**) correlation heatmap between oat quality parameters and oat metabolomics analysis. The colors red and blue represent positive and negative correlations, respectively. * *p* < 0.05 and ** *p* < 0.01.

As shown in Table S5, using the predefined criteria for differential metabolites (DFMs) (variable importance in projection [VIP] > 1 and *p* < 0.05), a total of 58 DFMs were detected in both positive and negative ion modes, with 31 in the positive ion mode and 27 in the negative ion mode. The KEGG pathway enrichment analysis shown in Figure 1A illustrates the top 12 pathways, with most related to pyrimidine, free amino acid, purine, and carbohydrate metabolism. A differential enrichment score was constructed to further compare the pathways associated with the DFMs leading to changes in feed metabolites between regions, as shown in Figure 1B.

The results in Table 4 show that 14 metabolic pathways were upregulated while 2 were downregulated in the GX group compared with the YX group (DA score > 0.5, *p* < 0.05). Butanoate metabolism; starch and sucrose metabolism; galactose metabolism; alanine, aspartate, and glutamate metabolism; ascorbic acid and aldolate metabolism; the TCA cycle; phenylalanine metabolism; the cAMP signaling pathway; taste transduction; GABAergic synaptic pathways; and fructose and mannose metabolism were among the metabolic pathways that were upregulated. On the other hand, purine and pyrimidine metabolism was downregulated. Succinate, 4-aminobutyric acid, sucrose, trehalose, raffinose, melibiose, D-allulose, D-galacturonic acid, D-arabinose, and L-arabinose-1,4-lactone were among the significant metabolites that were upregulated. In comparison, the downregulated metabolites included His-Lys, Ile-Pro, and His-Ser, as well as deoxyadenosine, deoxyguanosine, 2′-deoxycytidine, thymine, and cytosine.

Overall, carbohydrate and free amino acid metabolism were upregulated, while pathways associated with nucleotide metabolism were downregulated in the GX group compared with the YX group. This result indicates marked differences in the metabolic pathways in saline- and non-saline-grown oats.

#### 3.2.5. Correlation Between Oats Quality Parameters and Metabolomics

The correlation heatmap presented in Figure 1C illustrates the relationship between oat quality (nutritional quality, free amino acids, fatty acids, and sugars) and untargeted metabolites. Overall, there was a significant correlation between oat metabolites and oat quality. Specifically, RFV, crude protein, Ala, Met, Phe, Lys, Thr, Arg, Asn, Cho, Fru, Gal, Glu, Gly, Lev, c16:0, c16:1n7, c18:2n6, c18:3n3, c20:3n3, D-Ara, Mal, man, suc, tre, trehalose, xylose, and arabinitol were positively correlated with D-galactarate, D-allose, D-arabinose, raffinose, palatinose, sucrose, melibose, erythritol and succinate and negatively correlated with GABA, His-Ser, Ile-Pro, His-Lys, 5′-deoxyadenosine, deoxyguanosine, and palmitic acid.

### 3.3. Meat Quality Analysis

#### 3.3.1. Carcass Traits

Table 5 displays the differences in the carcass quality of Tibetan sheep meat between the YB and B0 groups. The carcass quality of Tibetan lamb meat did not differ significantly between the two groups, except for a significant difference in the area of the eye muscle, which was smaller in the animals belonging to the YB group than in the animals belonging to the B0 group (*p* < 0.05).

#### 3.3.2. Meat Sensory Evaluation Analysis

The results of the sensory scores of Tibetan sheep meat in the YB and B0 groups are shown in Table 5 and Figure S4. There were significant differences in the Tibetan sheep meat in the two groups (*p* < 0.05), apart from a lack of difference in meat color. The meat of animals belonging to the YB group scored higher than the B0 group in terms of aroma, juiciness, texture, and overall acceptability, indicating that meat from Tibetan sheep fed on saline oats had better sensory quality.

#### 3.3.3. Meat-Eating Quality Analysis

The results of the analysis of the eating quality of meat in groups YB and B0 are shown in Table 5. There were significant differences (*p* < 0.05) in the eating quality of the meat between the two groups in terms of thawing loss, cooking loss, cooked meat percentage, shear force, hardness, adhesion, and chewiness, with lower values for cooking loss, hardness, and chewiness in animals belonging to the YB group together with greater cooked meat percentage values relative to the animals belonging to the B0 group. No significant differences in the remaining indices, namely, pH, elasticity, and cohesion, were observed between the two groups.

#### 3.3.4. Meat Nutritional Quality Analysis

Table 5 indicates that the nutritional quality of the LL muscles of the Tibetan sheep in the two groups differed significantly (*p* < 0.05) in terms of protein and fat contents, with the meat of the animals belonging to the YB group having a greater protein content and lower fat content. However, there was no discernible variation in the groups’ moisture levels.

#### 3.3.5. Targeted Metabolomics Analysis of Meat

##### Free Amino Acid Analysis of Meat

As shown in Table 5, the free amino acid composition of the LL muscle of housed Tibetan sheep from saline and non-saline areas showed significant differences in the levels of most free amino acids (*p* < 0.05), with arginine, proline, creatine, glycine, leucine, isoleucine, creatinine, and valine being higher in the animals belonging to the YB group than those in the animals belonging to the B0 group. On the other hand, animals belonging to the YB group had lower levels of threonine, asparagine, and phenylalanine than animals belonging to the B0 group. Still, animals belonging to the YB group had significantly higher levels of NEAAs and TAAs than the B0 group (*p* < 0.05).

##### Fatty Acid Analysis of Meat

As shown in Table 5, the contents of most fatty acids in the LL muscles differed significantly between the two groups, with animals belonging to the YB group having lower levels of c15:0, c15:1n5, c20:5n3, c22:0, c22:1n9, c10:0, c18:3n6, c20:1n9, c20:3n6, and c20:4n6 than animals belonging to the B0 group. The levels of c16:0, c22:5n3, c16:1n7, c20:4n6, and c22:4n6 were higher than those in animals belonging to the B0 group (*p* < 0.05). In animals belonging to the YB group, the content of EPA was significantly lower than that in animals belonging to the B0 group (*p* < 0.05). There were no significant differences in PUFA, SFA, omega-3, omega-6, or total fatty acids.

#### 3.3.6. Untargeted Metabolomics Analysis of Tibetan Sheep Meat

##### Quality Control Analysis

The comparison of the spectrum overlap of the TICs for the QC samples is presented in the Supplementary Materials, Figure S5A,B. The peaks’ response intensities and retention periods significantly overlapped, suggesting negligible fluctuation attributable to instrumental error during the experiment. The peak regions from all the experimental and QC samples were then evaluated using PCA, as shown in the Supplementary Materials, Figure S6A,B. As seen in the figures, the QC samples were closely clustered in positive and negative ion modes, indicating the good reproducibility of the experiment. After excluding the QC samples, further evaluations using PLS-DA and OPLS-DA were performed to differentiate the samples further. Groups YB and B0 displayed intra-group clustering and inter-group separation in both analyses, with OPLS-DA demonstrating a more significant effect, as shown in the Supplementary Materials, Figure S6C,D. The replacement test was used to verify the supervised model’s validity to prevent overfitting during the modeling phase. Figure S6E,F in the Supplementary Materials show the replacement test plots of the OPLS-DA model, with a gradual decrease in the replacement retention and R^2^ and Q^2^ of the stochastic model, indicating an absence of overfitting in the original model with good stability.

##### Bioinformatics Analysis of Differential Metabolites in Meat

**Figure 2:**
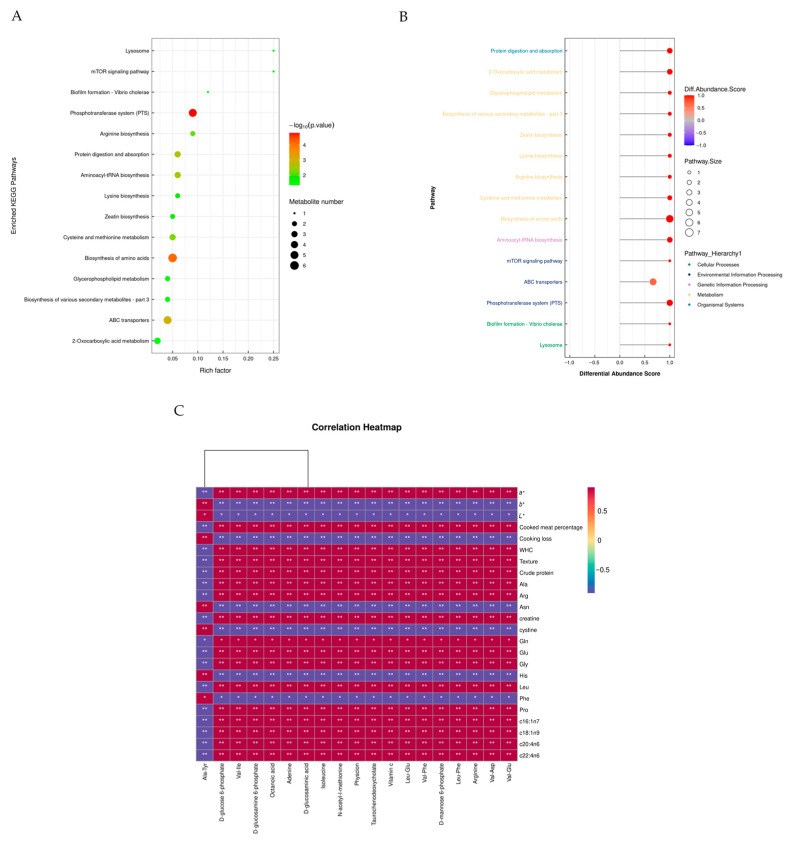
(**A**) Top 12 enriched KEGG pathways of the comparison between B0 and YB groups, (**B**) a differential abundance score map of differential metabolic pathways, (**C**) correlation heatmap between meat quality parameters and meat metabolomics analysis. The colors red and blue represent positive and negative correlations, respectively. * *p* < 0.05 and ** *p* < 0.01.

Overall, after database matching and secondary mass spectrometry verification, a total of 1133 effectively annotatable metabolites were obtained in the metabolomics experiments of this study, which were found in animals belonging to the YB group and the B0 group through a combination of positive and negative ion modes, with 632 identified in the positive ion mode and 501 in the negative ion mode. As shown in Table S6, differential metabolites were identified using the criteria of VIP > 1 and *p* < 0.05, finding 73 differential metabolites between groups Y and B0, of which 35 metabolites were identified in the positive ion mode and 38 in the negative ion mode; of these, 11 were enriched in KEGG pathways. The most significantly enriched pathways for free amino acid and lipid metabolism, as well as protein digestion and absorption, are presented in the KEGG pathway enrichment analysis in Figure 2A. To further compare the differential metabolite-associated pathways that may cause variations in feed metabolites across regions, various enrichment score plots were developed, as seen in Figure 2B.

The results in Table 6 show a marked upregulation of all metabolic pathways in animals belonging to the YB group compared with the B0 group (DA score > 0.5, *p* < 0.05). These pathways involved fructose and mannose metabolism, carbohydrate digestion and absorption, aminoacyl-tRNA biosynthesis, the mTOR signaling pathway, the phosphotransferase system, and lipid and free amino acid metabolism. The key upregulated metabolites included D-mannose-6-phosphate, D-glucose-6-phosphate, 2,6-diaminohexanoic acid, arginine, isoleucine, lysine, cysteine, and choline phosphate.

#### 3.3.7. Correlation Analysis

Correlations between the meat phenotypic data and the untargeted metabolomics results were established to investigate the relationship between muscle metabolism and meat quality in Tibetan sheep meat under various feeding conditions, as illustrated in Figure 2C.

The metabolites in the meat included D-glucose 6-phosphate, Val-Ile, D-glucosamine 6-phosphate, octanoic acid, adenine, D-glucosaminic acid, isoleucine, N-acetyl-l-methionine, physcion, taurochenodeoxycholate, vitamin C, Leu-Glu, Val-Phe, D-mannose 6-phosphate, Leu-Phe, arginine, Val-Asp, and Val-Glu, which were positively correlated with a*; the cooked meat percentage; WHC; texture; crude protein; and Ala, Arg, Gln, Glu, and Gly and negatively correlated with b*, L*, cooking loss, Asn, cystine, His, and Phe.

### 3.4. Relationship Between Quality and Metabolites of Oats Grown in Saline Soil and Quality and Metabolites of Tibetan Sheep Meat

**Figure 3:**
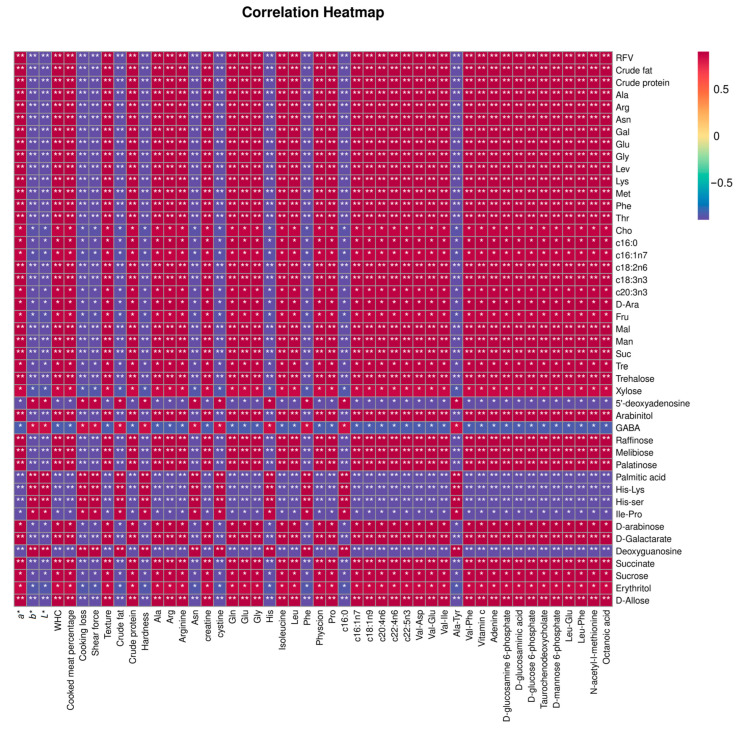
Clustering heatmap of correlation between metabolites and quality of oats and meat. * *p* < 0.05 and ** *p* < 0.01.

Figure 3 presents a correlation clustering heatmap that depicts the relationship between oat quality, metabolites, and the quality of Tibetan sheep meat and its metabolites. The figure illustrates a significant correlation between the two groups. The horizontal axes denote meat quality and associated metabolites, while the vertical axes represent oat quality and its metabolites.

Positive correlations were observed between crude protein, crude fat, Ala, Arg, Asn, Gal, Glu, Gly, Lev, Lys, Met, Phe, Thr, Man, Suc, Tre, trehalose, xylose, raffinose, melibiose, palatinose, succinate, sucrose, erythritol, and D-allose in oats and a*, WHC, cooked meat percentage, crude protein, Ala, Arg, Asn, vitamin C, adenine, D-glucosamine 6-phosphate, D-glucosaminic acid, D-glucose 6-phosphate, taurochenodeoxycholate, D-mannose 6-phosphate, and octanoic acid in meat. At the same time, these were negatively correlated with b*, L*, cooking loss, and shear force. These associations offer insights into the correlation between oat composition and muscle characteristics.

## 4. Discussion

In this study, it was found that the relatively high contents of mineral elements such as Ca, K, Mg, and Na in saline–alkaline soils may enhance the stress resistance of oats, enabling them to adapt to saline–alkaline environments and thus accumulate more osmotic regulatory substances to improve their quality. This may be because when salt concentration increases, ion imbalance and osmotic stress in plants can affect their morphology, biomass, and biochemical processes. Therefore, plants need to maintain a concentration of mineral elements within a specific range to achieve optimal physiological functions [25]. Moreover, changes in the availability of mineral contents in the soil can affect plant growth and quality. Under saline–alkaline stress, plants will eliminate excessive Na^+^ and Cl^−^ ions in vacuoles or older parts to minimize damage caused by excessive salt ions. Meanwhile, plants will biosynthesize osmotic substances, such as soluble sugars and amino acids, and activate enzymatic and non-enzymatic antioxidant defense systems to remove excess reactive oxygen species (ROS), protecting plant cells from oxidative damage [26]. Salt-tolerant plants may selectively absorb elements such as Ca, K, and Mg from the soil under saline–alkaline stress, increasing their mineral content and thus enhancing their nutritional value. The accumulation of Mg and Ca is beneficial for maintaining ion balance and regulating plant growth in saline–alkaline environments. Moreover, K can enhance plant stress resistance, strengthen cellular responses to adverse environmental conditions, and improve plant tolerance to challenging conditions such as saline–alkaline stress and drought [27]. Jin et al. [28] confirmed that the addition of potassium fulvate (PF), an organic fertilizer, to soil can alleviate nutritional antagonism and osmotic stress in oats under saline–alkaline conditions; increase the concentrations of total nitrogen, total potassium, and their available forms (ammonium nitrogen and nitrate nitrogen) in the soil; enhance the absorption of water and essential mineral nutrients by plants; and promote the growth of oats.

The use of male sheep as experimental subjects usually arises from the necessity to regulate variables, streamline the experimental framework, or investigate male-specific issues. This strategy requires careful evaluation of specific study aims, with careful consideration of the potential influence of gender variations on the credibility of scientific conclusions. This experiment exclusively employed male sheep, which presents limitations. In forthcoming trials, we aim to thoroughly examine the variations in meat quality among female sheep, male sheep, and castrated rams.

In this experiment, Tibetan sheep were raised in Gonghe and Haiyan. Geographical differences may potentially introduce the influence of environmental variables, such as temperature, humidity, and altitude. However, both regions belong to the alpine area of the Qinghai–Tibet Plateau, with similar climatic and altitude characteristics, and the purpose of this study is to clarify the differences between forage and meat quality. Therefore, in our study design, artificially adjustable variables were controlled, and group differences were solely established through roughage (oats from different regions), with the concentrate composition being identical between the two groups. Oats were harvested in the same growing season to avoid the impact of seasonality on nutrient accumulation. All meat quality analyses and metabolomics detections were performed in the same laboratory, eliminating inter-laboratory systematic errors. These measures reduced the influence of natural environmental differences. Future studies will further exclude the interference of environmental variables through cross-location feeding experiments to verify the robustness of the conclusions of this study.

In this study, the fat content of oats grown in saline conditions (GX group) was significantly higher than that of the non-saline oats (YX group). Li et al. [29] conducted a lipidomic analysis of salt-treated alfalfa varieties. They found that the lipid levels in the plants changed under salt stress, indicating that salt treatment affected the plants’ fat and fatty acid contents. This may have been due to changes in the structural integrity and fluidity of cell membranes in plant leaves during salt stress. Ge et al. [30] found that the content of galactolipids (DGDG and MGDG) and major phospholipids (PC and PE) increased in the leaves of sorghum seedlings under salt stress; this is likely due to salt stress-induced changes in the glycerolipid pathway between the cytoplasm and plastid, facilitating the conversion of PC to PA and providing precursors for galactolipid synthesis. According to the study, herbage on saline–alkaline land had a higher concentration of fatty acids (including SFA, PUFA, n-3, and n-6) than non-saline–alkaline land. This finding may be directly related to saline–alkaline stress.

Free amino acids are stress-response metabolites that accumulate under saline and alkaline stress; this accumulation may result from either de novo synthesis or protein degradation, accelerating post-stress recovery and osmoprotection [31]. Qian et al. [32] demonstrated that under saline–alkaline stress, the amino acid content increases significantly, and amino acid biosynthesis exhibits a positive response to saline–alkaline stress. The current study’s findings indicated that serine, valine, glycine, arginine, and alanine concentrations were elevated in the GX group compared with the YX group, potentially linked to salinity stress. Untargeted metabolomics results further demonstrated upregulation in alanine, aspartate, glutamate, and phenylalanine metabolism, aligning with the targeted metabolism outcomes for free amino acids. Furthermore, correlation analysis suggested that the accumulation of glutamate and succinate may have enhanced the salinity tolerance of the oat grasses in the YX group. Proteomic analysis of saline alfalfa by Gao et al. [33] revealed that key differentially expressed proteins were primarily enriched in the antioxidant system and starch and sucrose metabolism, as well as in secondary metabolism, suggesting that saline and alkaline stress increases antioxidant functions in the plants together with the production of secondary metabolites, such as sucrose, maltose, glucose, and trehalose, and the promotion of osmotic homeostasis. This study’s untargeted metabolomics and subsequent KEGG analysis indicated that starch and sucrose metabolism were upregulated, resulting in increased carbohydrate buildup. Starch is the primary carbohydrate in oats and a vital element, significantly contributing to the structural integrity of oat grains. As described by Rostamabadi et al. [34], the resistant starch in oat starch can bind to bile acids, preventing their reabsorption, promoting the conversion of cholesterol into other bile acids, and maintaining the balance of bile acid excretion. Consequently, this process reduces the cholesterol level in the blood, which benefits human health.

Overall, in the nutritional quality and metabolomics analysis of saline-grown and non-saline-grown oats, the GX saline oats group showed significant advantages as ruminant feed.

Despite significant differences in meat quality and composition among animals in different dietary groups, there were no statistical differences in growth performance, such as growth rate and final weight. It is hypothesized that the dietary formulation in the experiment generated differences between groups solely through roughage (oats from different regions), with the concentrate composition being identical between the two groups, all of which met the basic nutritional requirements for animal growth. Therefore, no differences were observed in the overall growth indicators, while dietary differences may mainly affect meat quality by regulating muscle metabolic pathways. Moreover, the sheep involved in the experiment were all of the same breed, and the consistency in their genetic background and growth stage contributed to the stability of growth characteristics and some carcass indicators [35].

The tenderness of meat determines consumer acceptability and satisfaction. The shear force value of the meat in group YB was less than that of group B0, indicating more significant tenderness. The sensory evaluations suggested that the sheep muscles from animals belonging to the YB group showed improved texture. Correlation analysis revealed a positive relationship between muscle texture and water-holding capacity (WHC) with D-glucose-6-phosphate. The upregulation of D-glucose-6-phosphate in animals belonging to the YB group may enhance glycolysis by modulating starch and sucrose metabolic pathways, resulting in ATP production and degradation of glycogen. This process likely leads to a rapid decrease in pH, facilitating the hydrolysis of fibrous muscle proteins and improving muscle tenderness, which may account for the increased tenderness observed in animals belonging to the YB group. Muscle tenderness is influenced by factors including collagen content, knob length, and protein degradation [36]. The present study found that the contents of free glutamic acid and glycine in the muscles of Tibetan sheep fed with oats from saline soils were significantly increased, accompanied by a decrease in shear force value, improvement in tenderness, and an increase in aroma. This may be because glutamic acid, as a key substrate for muscle protein synthesis, can promote the degradation of collagen between muscle fibers through its accumulation, thereby reducing the degree of cross-linking between muscle fibers, decreasing shear force, and improving tenderness. Meanwhile, as synergistic activators of umami receptors, glutamic acid and glycine can directly enhance taste signal transmission and improve the umami perception of meat quality with their increased contents [37]. Tibetan sheep fed with saline oats had increased levels of linoleic acid in their muscles, along with an improved water-holding capacity score. This may be due to the fact that linoleic acid, as an n-6 polyunsaturated fatty acid, can lower the melting point of muscle fat, making it easier for fat to melt during oral mastication and release flavor substances, thereby enhancing the perception of juiciness. Meanwhile, linolenic acid can reduce the production of fat oxidation products by inhibiting the activity of muscle lipoxygenase, avoiding the dry and hard taste caused by fat oxidation, and indirectly maintaining juiciness. The positive correlation between these two fatty acids and juiciness indicators further verifies the regulatory effect of fatty acid composition on meat juiciness [38].

Generally, higher a* and lower L* and b* values indicate better muscle color within a specific range. In this study, animals belonging to the YB group had higher a* values, with the meat showing a brighter red color than that in the B0 group, which may be related to the production of oxygenated myoglobin. It has been reported that L* values are positively associated with muscle WHC; water exudation causes changes in the refractive index of the muscle surface, resulting in a higher L* value [33]. In the present study, animals belonging to the YB group showed better WHC values, indicating less water exudation from the muscle and, thus, a lower L* value. The lower cooking loss and higher cooked meat rate in animals belonging to the YB group in this study may have been due to the changes in the structure of the muscle fibers, leading to the improved water retention capacity of the muscle proteins [39]. The results of the correlation study showed that D-glucose-6-phosphate was negatively connected with cooking loss and the L* value and significantly and positively correlated with the a* value, cooked meat rate, WHC, and texture. This suggests that glycolysis controls the quality of meat. Collectively, saline-region Tibetan lamb showed improved eating and sensory characteristics.

In the present study, it was found that Tibetan sheep meat in the YB group had higher protein and lower fat contents, which is similar to the findings of Friha et al. [40], who reported that rearing lambs on saline soils resulted in better growth performance, leaner carcasses, and higher meat quality. This result may be related to the composition and proportions of free amino acids and fatty acids. Free amino acids are the building blocks of muscle proteins, and their contents and compositions affect meat texture, flavor, nutritional value, and antioxidant capacity [41]. Leucine, isoleucine, and valine, collectively known as branched-chain free amino acids (BCAAs) due to their characteristic side-chain structures and specific metabolic pathways, promote protein synthesis and tissue growth by stimulating mRNA translation through mTORC1 signaling [42]. In addition to encouraging the release of prolactin, growth hormone, and insulin from the corresponding endocrine organs, arginine enhances the expression of essential proteins and enzymes involved in substrate oxidation and mitochondrial biosynthesis, controlling muscle oxidation and lowering excess body fat in animals [43]. Proline acts as a substrate and is involved in the synthesis of pyruvate and glucose, which is associated with the collagen content of muscles. At the same time, threonine increases the meat’s sweetness, making it more palatable to consumers [44]. In this study, animals belonging to the YB group were associated with increased proline, serine, and lysine deposition in the LL muscles through upregulation of free amino acid metabolism. Compared with animals belonging to the YB group, animals in the B0 group were associated with reduced deposition of branched-chain free amino acids in the muscle by downregulating leucine, isoleucine, and valine biosynthesis. Furthermore, by upregulating the metabolism of creatinine and arginine, animals belonging to the YB group were able to collect higher quantities of arginine. Therefore, it is anticipated that the meat from animals belonging to the YB group raised in saline conditions has free amino acid concentrations that are more in accordance with what is required for human health.

Glycogen is the primary source of energy for the production of glycolytic substrates in postmortem muscle when the electron transport chain is terminated due to hypoxia; pyruvate is unable to enter the mitochondria, and the muscle accumulates lactic acid and hydrogen ions to maintain homeostasis, resulting in the denaturation of muscle proteins and a decrease in pH [45]. The phosphotransferase system (PTS) re-reduces glucose phosphorylation through phosphorylation and carbon source transfer, with associated enzymes hydrolyzing intracellular cAMP and lowering its concentration. This lowers glycogen metabolism by blocking the cAMP-dependent protein kinase (APK) signaling pathway and phosphorylase activity. AMPK, the primary regulator of lipid and glucose metabolism in the cell, is activated when the cell is depleted of energy [46]. Lamberigts et al. [47] found that intraperitoneal injection of α-lipoic acid inhibited AMPK activity in the hypothalamus, reducing food intake and energy expenditure. In the present study, PTS and α-lipoic acid metabolism were significantly upregulated in the animals belonging to the YB group compared with the B0 group, suggesting that glycogen metabolism was inhibited in this group. Hexokinase (HK), the first rate-limiting enzyme in the glycolytic pathway, phosphorylates glucose to generate D-glucose-6 phosphate (G6P); moreover, negative feedback involving intracellular G6P, a central regulator of glycogen synthesis in skeletal muscle, regulates HK [48]. Glucose-6-phosphate dehydrogenase (G6PD) catalyzes the conversion of G6P into glucose-6-phosphate-δ-lactone, which engages glucose in the pentose phosphate pathway to form NADPH. NADPH is a reducing agent that generates reduced glutathione, which helps to counteract oxidative stress and maintain cellular homeostasis and normal metabolic activities [49]. In the present study, the results of the untargeted metabolomics indicated that G6P levels were higher in animals belonging to the YB group than in the B0 group, accompanied by upregulated glutathione metabolism. The current study proposes that animals belonging to the YB group might show diminished glycolysis due to reduced HK activity and elevated glucose-6-phosphate dehydrogenase activity in the LL muscles of Tibetan sheep, thus sustaining a higher pH and enhancing water retention and inter-fiber bonding. This would yield meat with a denser structure, demonstrating enhanced tenderness and texture. At the same time, reduced oxygen diffusion from the muscle surface would enable greater absorption and lower light reflection, leading to a decrease in the muscle L*.

In high-altitude hypoxic regions, glucose metabolism produces NADPH via the pentose phosphate pathway (PPP), stabilizing the hypoxia-inducible factor HIF-1α protein [50]. Therefore, it was hypothesized that both the PP and HIF-1α pathways were significantly upregulated in animals belonging to the YB group. The PPP is a fundamental component of cellular metabolism. It is essential for maintaining carbon homeostasis, providing nucleotide and free amino acid biosynthesis precursors, supplying reducing molecules for antioxidant activities, and resisting oxidative stress [51]. HIF-1α regulates the activities of antioxidant enzymes such as glutathione peroxidase and heme oxygenase. Further, elevated expression of HIF-1α may also increase the expression of most antioxidant proteins in muscle tissues by enhancing the expression of Nrf2, increasing the antioxidant capacity of the organism [52]. Vitamin C, ascorbic acid, is a non-enzymatic antioxidant that effectively scavenges ROS [53]. Initially isolated from bovine bile, taurine is a sulfur-containing free amino acid that modulates the intracellular enzymatic antioxidant defense system by activating various signaling pathways and enhancing catalase activity in response to stress [54]. Compared with the B0 group, animals belonging to the YB group were associated with increased levels of vitamin C and taurine, resulting from the upregulation of bile secretion and vitamin digestion and absorption, leading to improved antioxidant activities in the muscle and thus improving the meat quality and nutritional value.

**Figure 4:**
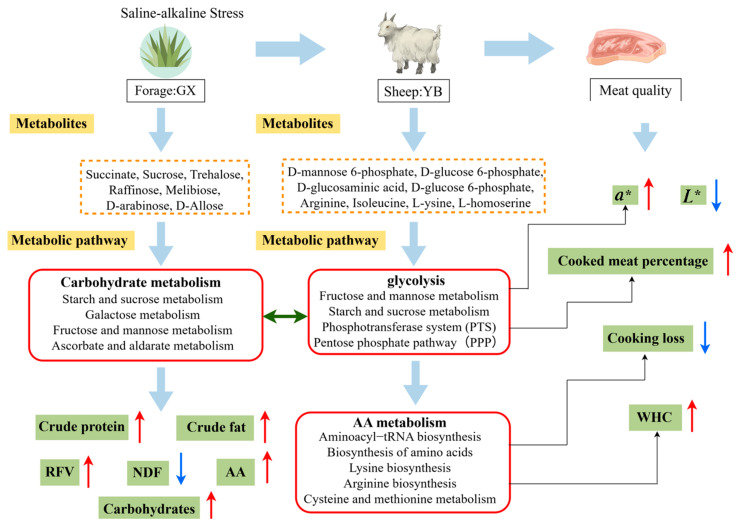
Hypothesized scheme pathways and potential mechanisms related to changes in oat quality, the muscle metabolome, and meat quality. Blue and red colors indicate significantly downregulated and upregulated metabolic pathways in each comparison, respectively. L* represents Lightness, a* represents Redness.

In summary, as shown in Figure 4, it is hypothesized that the saline oat YX group accumulated a large number of carbohydrates during adaptation to saline stress. As a result, the levels of carbohydrates such as D-glucose-6-phosphate and D-mannose-6-phosphate in the meat were significantly increased. The upregulation of these compounds resulted in enhanced activation of the PPP and PTS pathways in the LL muscles of the sheep. The abundance of G6P specifically blocks hexokinase activity in the upregulated PTS pathway, therefore postponing a decline in pH. This postponement in pH lowering enhances water retention in the muscle, diminishes cooking losses, elevates the percentage of cooked meat and water-holding capacity, and preserves meat quality.

Furthermore, the transfer of G6P to the PPP was improved by reducing hypoxia and the requirement to raise cellular antioxidant capacity by increasing NADPH production. The resulting NADPH further increased the muscle’s antioxidant capacity, thus lessening the harm that oxidative stress caused to the animals. Meanwhile, Tibetan lamb in saline areas had higher-quality meat because the upregulation of free amino acid pathways in saline oats resulted in the upregulation of free amino acid pathways in muscle, as well as the deposition of free amino acids in muscle.

## 5. Conclusions

Oats cultivated in saline–alkaline land affect the meat quality of Tibetan sheep. Specifically, Tibetan sheep in the YB group fed with saline oats exhibited better meat color, a higher cooked meat rate and water-holding capacity, and lower cooking loss. This resulted from the higher protein, monosaccharide, and amino acid contents in saline oats, which promoted the upregulation of various amino acids such as arginine and lysine in muscles; the digestion and absorption of carbohydrates such as D-glucose-6-phosphate and D-mannose-6-phosphate; and the accumulation of antioxidant components, including ascorbic acid and taurine. In conclusion, the increase in compatible solutes in saline oats had a significant effect on maintaining the WHC of Tibetan sheep muscles, improving tenderness, and enhancing sensory quality. Therefore, this finding provides a promising theoretical basis for optimizing meat sheep production and feeding management.

## Figures and Tables

**Table 1:** Diet composition and nutritional level (dry matter basis, %).

Dietary Composition	B0 (%)	YB (%)
Corn	27.6	27.6
Soybean meal	3.6	3.6
Canola meal	6.6	6.6
Cottonseed meal	9.6	9.6
Wheat	7.8	7.8
Sodium chloride	0.6	0.6
Limestone	0.6	0.6
Sodium bicarbonate	0.6	0.6
Premix	3	3
Total	60	60
Roughage	40 (Haiyan oats)	40 (Gonghe oats)

Note: YB represents feeding with oats grown in saline–alkali land, and B0 represents feeding with oats grown in non-saline–alkali land. The same below.

**Table 2:** Differences in mineral element contents between saline–alkali soils and non–saline–alkali soils (mg·kg^−1^).

Mineral Elements	Groups		*p*
	Saline-Alkali Soils	Non-Saline-Alkali Soils	
Al	68,035.35 ± 4736.96	34,008.04 ± 1424.82	<0.001
Ca	57,564.72 ± 209.07	13,656.04 ± 222.85	<0.001
Fe	26,630.92 ± 71.74	32,134.5 ± 44.50	<0.001
K	20,612.75 ± 182.08	16,377.34 ± 81.11	<0.001
Mg	13,457.35 ± 299.94	6690.74 ± 99.85	<0.001
Mn	691.79 ± 7.89	884.94 ± 6.80	<0.001
Na	15,369.20 ± 87.31	11,658.48 ± 5.98	<0.001
P	718.92 ± 1.80	1370.62 ± 26.10	<0.001
S	1071.58 ± 64.08	661.8 ± 28.56	<0.001
V	6.49 ± 0.30	99.45 ± 0.46	<0.001
Cr	65.84 ± 1.68	104.77 ± 0.85	<0.001
Co	0.86 ± 0.01	15.25 ± 0.10	<0.001
Ni	2.25 ± 0.19	38.21 ± 0.20	<0.001
Cu	15.56 ± 0.44	18.22 ± 0.28	<0.001
Zn	73.48 ± 0.13	109.20 ± 0.45	<0.001
Se	0.04 ± 0.01	0.50 ± 0.01	<0.001
Rb	5.49±0.35	18.97±0.82	<0.001

**Table 3:** Differences in oat quality between regions.

Items	Groups		*p*
	GX	YX	
Analysis of oat nutritional values			
Moisture (%)	73.94 ± 0.01	72.97 ± 0.01	0.520
Crude protein (%)	9.03 ± 0.02	7.16 ± 0.04	<0.001
Crude fat (g/kg)	14.92 ± 0.87	7.45 ± 0.88	0.005
Crude fiber (%)	6.37 ± 0.06	6.57 ± 0.15	0.139
NDF (%)	53.37 ± 0.38	57.30 ± 0.30	<0.001
ADF (%)	31.37 ± 0.24	32.33 ± 0.05	0.073
DMI (%)	2.25 ± 0.01	2.09 ± 0.01	0.010
DDM (%)	63.84 ± 0.19	63.07 ± 0.40	0.147
RFV (%)	111.28 ± 0.51	102.38 ± 0.49	0.001
Amino acid composition (mg/100 g)			
Serine	1130.88 ± 16.86	512.06 ± 1.32	<0.001
Valine	961.42 ± 10.77	549.16 ± 21.54	<0.001
Glycine	1178.60 ± 8.73	398.49 ± 29.40	0.002
Lysine	46.34 ± 0.53	34.43 ± 0.96	0.002
Arginine	1458.35 ± 95.18	613.77 ± 16.49	0.003
Alanine	208.71 ± 0.95	159.47 ± 3.46	0.004
Phenylalanine	1474.83 ± 87.32	749.18 ± 31.37	0.004
Tyrosine	368.95 ± 16.62	195.97 ± 3.90	0.004
Asparagine	20.99 ± 2.66	7.09 ± 0.85	0.006
Glutamate	238.90 ± 13.04	85.02 ± 20.90	0.009
Methionine	107.60 ± 7.53	53.66 ± 2.05	0.010
Spermidine	6.00 ± 0.73	2.82 ± 1.28	0.020
Choline	310.25 ± 25.01	231.43 ± 8.31	0.024
Citrulline	1.50 ± 0.32	0.51 ± 0.24	0.030
Threonine	1549.18 ± 50.01	1149.47 ± 79.41	0.031
Glutamine	0.27 ± 0.02	0.22 ± 0.01	0.040
Hydroxyproline	0.15 ± 0.01	0.13 ± 0.00	0.040
Aspartate	105.13 ± 2.35	68.86 ± 12.54	0.043
Aminoadipic Acid	0.30 ± 0.02	0.36 ± 0.01	0.044
Creatinine	0.01 ± 0.00	0.01 ± 0.00	0.070
Histidine	224.10 ± 10.95	198.97 ± 24.16	0.072
Proline	387.29 ± 10.60	353.17 ± 10.22	0.091
Isoleucine	445.08 ± 27.86	402.20 ± 3.80	0.120
Creatine	0.08 ± 0.00	0.29 ± 0.18	0.150
Leucine	412.86 ± 33.75	384.23 ± 3.80	0.220
Taurine	0.06 ± 0.01	0.18 ± 0.09	0.220
Tryptophan	4.44 ± 1.13	2.88 ± 0.56	0.340
Ornithine	0.49 ± 0.08	0.44 ± 0.14	0.410
Putrescine	1.49 ± 0.16	1.85 ± 1.11	0.690
Cystine	4.74 ± 2.89	5.21 ± 2.85	0.910
Cysteine	0.03 ± 0.00	0.03 ± 0.01	0.950
EAA	5118.25 ± 173.30	3470.51 ± 52.00	0.012
NEAA	5530.76 ± 131.29	2691.04 ± 58.33	0.002
TAA	10,649.00 ± 214.13	6161.56 ± 106.44	0.003
Fatty acid composition (mg/100 g)			
Decanoic acid (c10:0)	0.01 ± 0.00	0.01 ± 0.00	0.001
Heneicosanoic acid (c21:0)	0.09 ± 0.01	0.17 ± 0.01	0.002
Nervonic acid (c24:1n9)	0.17 ± 0.01	0.25 ± 0.00	0.006
Eicosenoic acid (c20:1n9)	0.56 ± 0.10	1.01 ± 0.04	0.010
Linoleic acid (c18:2n6)	41.49 ± 4.66	23.61 ± 0.33	0.018
Docosapentaenoic acid (c22:5n3)	0.05 ± 0.00	0.06 ± 0.01	0.019
Palmitoleic acid (c16:1n7)	4.72 ± 0.41	2.71 ± 0.35	0.021
Heptadecanoic acid (c17:0)	3.53 ± 0.55	1.89 ± 0.38	0.022
Eicosatrienoic acid (c20:3n3)	0.25 ± 0.00	0.32 ± 0.02	0.027
Arachidic acid (c20:0)	0.33 ± 0.04	0.23 ± 0.01	0.028
α-Linolenic acid (c18:3n3)	1.09 ± 0.17	0.77 ± 0.05	0.035
Palmitic acid (c16:0)	259.90 ± 19.93	156.37 ± 20.82	0.042
Eicosadienoic acid (c20:2n6)	0.16 ± 0.05	40.63 ± 1.27	0.060
Eicosapentaenoic acid (c20:5n3)	0.70 ± 0.07	0.07 ± 0.01	0.134
Lignoceric acid (c24:0)	0.90 ± 0.08	0.69 ± 0.04	0.178
Pentadecenoic acid (c15:1n5)	0.61 ± 0.02	0.79 ± 0.07	0.180
Pentadecanoic acid (c15:0)	0.14 ± 0.02	0.80 ± 0.15	0.195
Heptadecenoic acid (c17:1n7)	0.06 ± 0.02	0.14 ± 0.00	0.232
Myristoleic acid (c14:1n5)	0.09 ± 0.03	0.05 ± 0.01	0.260
Docosadienoic acid (c22:2n6)	0.05 ± 0.01	0.12 ± 0.01	0.318
Tricosanoic acid (c23:0)	0.13 ± 0.02	0.05 ± 0.00	0.380
Myristic acid (c14:0)	0.92 ± 0.22	0.16 ± 0.02	0.440
Lauric acid (c12:0)	0.51 ± 0.14	0.72 ± 0.13	0.451
Octanoic acid (c8:0)	0.01 ± 0.00	0.45 ± 0.05	0.480
Tridecanoic acid (c13:0)	0.01 ± 0.00	0.01 ± 0.00	0.570
Stearic acid (c18:0)	3.75 ± 0.39	0.01 ± 0.01	0.600
Docosahexaenoic acid (c22:6n3)	0.13 ± 0.02	4.11 ± 0.77	0.930
ΣPUFA	302.81 ± 13.24	181.21 ± 17.73	0.020
ΣSFA	55.37 ± 1.22	48.29 ± 1.57	0.040
ΣPUFA: ΣSFA	5.48 ± 0.35	3.77 ± 0.47	0.062
ΣMUFA	7.73 ± 0.05	6.83 ± 0.55	0.100
Σn-3	261.11 ± 17.30	157.49 ± 18.02	0.035
Σn-6	41.71 ± 4.06	23.72 ± 0.29	0.018
EPA	0.70 ± 0.06	0.69 ± 0.03	0.452
DHA	0.13 ± 0.02	0.14 ± 0.02	0.466
TFA	365.91 ± 12.09	236.33 ± 16.91	0.016
Carbohydrate content (mg/100g)			
Sucrose	4643.76 ± 78.41	1787.41 ± 158.78	<0.001
Levoglucosan	4.55 ± 0.03	3.45 ± 0.02	0.001
Mannose	4.81 ± 0.09	3.35 ± 0.13	0.002
Xylulose	1.18 ± 0.01	0.93 ± 0.01	0.003
Fructose	2934.81 ± 51.76	2743.11 ± 38.97	0.003
Glucuronic Acid	1.22 ± 0.03	1.45 ± 0.02	0.006
Galactose	43.86 ± 0.74	40.51 ± 0.44	0.012
Arabinitol	3.31 ± 0.37	1.61 ± 0.01	0.014
Maltose	61.09 ± 5.51	32.99 ± 0.83	0.015
Galacturonic Acid	0.85 ± 0.08	1.12 ± 0.09	0.019
D-Arabinose	4.33 ± 0.21	3.56 ± 0.06	0.026
Xylose	4.21 ± 0.16	3.71 ± 0.09	0.029
Trehalose	10.91 ± 0.78	8.47 ± 0.12	0.036
Ribose	8.56 ± 0.40	7.57 ± 0.20	0.036
2-Ace-2-Deo-D-Glucosamine	1.46 ± 0.05	1.63 ± 0.08	0.093
Cellobiose	44.77 ± 6.96	36.76 ± 3.10	0.121
Rhamnose	1.49 ± 0.26	1.22 ± 0.00	0.164
Inositol	32.38 ± 3.19	29.04 ± 3.79	0.183
Ribose-5-pho-Ba	1.34 ± 0.13	1.80 ± 0.34	0.198
Phenylalanine	3.51 ± 0.47	3.14 ± 0.18	0.516
Glutamic Acid	4715.67 ± 130.46	4025.62 ± 189.39	0.704
Xylitol	0.76 ± 0.03	0.75 ± 0.05	0.756
2-Deoxyribose	0.62 ± 0.02	0.61 ± 0.01	0.785
Fucose	2.06 ± 0.13	2.10 ± 0.14	0.820
Sorbitol	2.68 ± 0.53	2.71 ± 0.21	0.956

Note: *p* < 0.05 and *p* < 0.01 compared with the YX group. GX represents oats grown on saline–alkaline land, and YX represents oats grown in non-saline–alkaline land. The same below. NDF represents neutral detergent fiber, ADF represents acid detergent fiber, DMI represents dry matter intake, DDM represents digestible dry matter, RFV represents relative feeding value. EAA = sum of valine, lysine, phenylalanine, methionine, leucine, isoleucine, tryptophan, histidine, and threonine; NEAA = sum of serine, glycine, arginine, alanine, tyrosine, asparagine, glutamic acid, spermidine, choline, citrulline, glutamine, hydroxyproline, aspartic acid, proline, cysteine, cystine, and taurine; TAAs = total amino acids. ΣSFA: total saturated fatty acids; ΣMUFA: total monounsaturated fatty acids; ΣPUFA: total polyunsaturated fatty acids; Σn-3: total omega-3 PUFA; Σn-6: total omega-6 PUFA; EPA: eicosapentaenoic acid (C20:5n-3); DHA: docosahexaenoic acid (C22:6n-3). Saturated fatty acids include C6:0, C8:0, C10:0, C11:0, C12:0, C13:0, C14:0, C15:0, C16:0, C17:0, C18:0, C20:0, C21:0, C23:0, C24:0. Polyunsaturated fatty acids include C18:2n6, C18:3n3, C20:2n6, C20:3n3, C20:5n3, C22:2n6, C22:5n3, C22:6n3. Monounsaturated fatty acids include C14:1n5, C15:1n5, C16:1n7, C17:1n7, C18:1n9, C24:1n9. Total n-3 fatty acids include C18:3n3, C20:3n3, C20:5n3, C22:5n3, C22:6n3. Total n-6 fatty acids include C18:2n6, C20:2n6, C22:2n6. TFA represents total fatty acids.

**Table 4:** DFMs (differential metabolites) from oats in the key differential enriched KEGG pathways (false discovery rates < 0.05).

Metabolic Pathways (GX vs. YX)	Metabolites
Upregulation in the GX group	
cAMP signaling pathway	Succinate, 4-Aminobutyric acid (GABA)
Taste transduction	Sucrose, Saccharose
	4-Aminobutyric acid (GABA)
GABAergic synapse	Succinate, Butanedionic acid
	4-Aminobutyric acid (GABA)
	Ethylenesuccinic acid
Butanoate metabolism	Succinate, Succinic acid Butanedionic acid Ethylenesuccinic acid
	4-Aminobutyricacid (GABA) gamma-Aminobutyric acid
Starch and sucrose metabolism	Trehalose, Sucrose
	1-α-D-Glucopyranosyl-2-β-D-fructofuranoside
Galactose metabolism	Sucrose, Cane sugar
	Raffinose, Saccharose, Melibiose 1-α-D-Glucopyranosyl-2-β-D-fructofuranoside
Alanine, aspartate, and glutamate metabolism	Succinate, Butanedionic acid Ethylenesuccinic acid
	gamma-Aminobutyric acid
Ascorbate and aldarate metabolism	D-Galactarate, D-Mucic acid
	D-arabinose
	L-Arabinono-1,4-lactone
Biosynthesis of secondary metabolites	Trehalose
Citrate cycle (TCA cycle)	Succinate, Butanedionic acid Ethylenesuccinic acid
Phenylalanine metabolism	Succinate, Butanedionic acid Ethylenesuccinic acid
Pyruvate metabolism	Succinate, Butanedionic acid Ethylenesuccinic acid
Amino sugar and nucleotide sugar metabolism	D-arabinose, L-Arabinose, L-Arabinopyranose
Fructose and mannose metabolism	D-Allose
Downregulation in the GX group	
Purine metabolism	Deoxyguanosine, His-Lys
	Deoxyadenosine
Pyrimidine metabolism	Ile-Pro, His-ser, 5-Methyluracil
	Thymine, Cytosine, Cytidine
	2′-deoxycytidine, Deoxythymidine

**Table 5:** The effect of different regional oats on carcass quality of Tibetan sheep meat.

Items	Groups		*p*
	YB	B0	
Carcass quality			
Average daily feed intake (kg)	1.45 ± 0.05	1.41 ± 0.08	0.441
Remaining after the feeding (kg)	0.63 ± 0.06	0.71 ± 0.08	0.494
Refuse percentage (%)	30.13 ± 0.02	33.46 ± 0.04	0.046
Initial weight (kg)	15.73 ± 0.88	16.14 ± 0.65	0.316
Final weight (kg)	34.55 ± 0.79	35.84 ± 1.23	0.154
Rib fat thickness (cm)	1.56 ± 0.70	1.53 ± 0.23	0.800
Abdominal fat thickness (cm)	1.79 ± 0.14	1.89 ± 0.17	0.360
Backfat thickness (cm)	1.57 ± 0.12	1.60 ± 0.15	0.620
Eye muscle area (cm^2^)	17.20 ± 1.64	26.00 ± 4.90	0.010
Edible quality			
Color	6.89 ± 1.20	6.00 ± 1.59	0.255
Aroma	7.78 ± 0.97	6.11 ± 1.45	0.011
Juiciness	7.89 ± 1.45	6.11 ± 1.30	0.013
Taste	8.22 ± 1.10	6.67 ± 1.41	0.019
Texture	8.44 ± 0.73	6.22 ± 1.56	0.001
General acceptability	8.44 ± 0.88	7.00 ± 1.41	0.019
pH45min	6.16 ± 1.08	6.72 ± 0.28	0.080
pH24h	5.91 ± 0.34	5.82 ± 0.43	0.510
L*	32.79 ± 0.57	36.50 ± 0.97	0.001
a*	21.11 ± 0.95	16.89 ± 1.79	0.002
b*	7.91 ± 0.83	10.57 ± 0.97	0.004
Thawing loss (%)	6.94 ± 2.38	7.80 ± 1.11	0.001
Cooking loss (%)	32.15 ± 4.79	52.25 ± 4.79	0.001
Cooked meat percentage (%)	67.67 ± 1.86	32.94 ± 1.23	0.003
water holding capacity (%)	15.70 ± 1.84	13.78 ± 1.85	0.036
Shear force (N)	130.14 ± 18.66	177.99 ± 16.50	0.020
Hardness (g)	11.00 ± 9.31	38.37 ± 32.58	0.010
Elasticity (mm)	2.62 ± 0.43	3.07 ± 0.54	0.160
Adhesion (g)	5.04 ± 3.90	22.52 ± 20.67	0.024
Chewability (mJ)	17.75 ± 2.93	37.86 ± 6.67	0.015
Cohesion (g)	0.46 ± 0.09	0.51 ± 0.14	0.312
Moisture (%)	74.45 ± 0.01	70.97 ± 0.04	0.348
Protein (%)	22.69 ± 0.36	19.80 ± 0.42	0.023
Fat (%)	0.60 ± 0.07	0.80 ± 0.07	0.001
Amino acid composition (mg/100 g)			
Arginine	1365.11 ± 15.35	260.31 ± 11.81	<0.001
Threonine	253.24 ± 0.57	774.26 ± 14.94	<0.001
Proline	236.12 ± 3.56	168.11 ± 1.79	<0.001
Asparagine	2.94 ± 0.05	6.03 ± 0.07	<0.001
Histidine	280.19 ± 8.70	1015.98 ± 23.55	0.001
Cystine	27.91 ± 3.13	314.68 ± 14.38	0.001
Phenylalanine	396.48 ± 37.01	551.87 ± 28.18	0.001
Glycine	1061.81 ± 15.46	739.96 ± 5.44	0.001
Hydroxyproline	3.40 ± 0.18	2.02 ± 0.08	0.002
Alanine	315.43 ± 1.14	297.18 ± 1.31	0.002
Leucine	580.62 ± 17.07	250.85 ± 14.56	0.003
Lysine	610.88 ± 36.57	324.15 ± 21.30	0.004
Isoleucine	383.43 ± 13.07	203.85 ± 13.79	0.007
Citrulline	4.86 ± 0.41	2.60 ± 0.39	0.008
Creatinine	2.41 ± 0.24	0.86 ± 0.10	0.008
Taurine	36.16 ± 3.11	14.13 ± 0.78	0.008
Valine	388.26 ± 16.68	216.45 ± 11.02	0.008
Glutamate	153.96 ± 10.37	62.13 ± 8.04	0.010
Creatine	125.90 ± 10.35	60.28 ± 2.06	0.013
Aminoadipic Acid	0.47 ± 0.06	0.21 ± 0.04	0.015
Cysteine	0.06 ± 0.01	0.23 ± 0.05	0.024
Glutamine	19.03 ± 3.11	8.38 ± 0.33	0.029
Methionine	45.60 ± 7.22	92.40 ± 10.33	0.032
Tryptophan	1.92 ± 0.04	3.25 ± 0.44	0.038
Aspartate	101.43 ± 7.93	80.51 ± 0.00	0.042
Serine	270.82 ± 2.33	285.00 ± 7.57	0.060
Tyrosine	336.99 ± 14.85	293.83 ± 10.74	0.087
Ornithine	0.32 ± 0.02	0.28 ± 0.02	0.157
Choline	332.25 ± 16.98	317.66 ± 20.87	0.342
EAA	2940.62 ± 20.23	3433.06 ± 33.55	0.004
NEAA	4397.37 ± 54.82	2914.39 ± 24.00	0.002
TAA	7337.99 ± 64.91	6347.45 ± 55.70	0.001
Fatty acid composition (mg/100 g)			
Pentadecanoic acid (c15:0)	1.63 ± 0.08	4.84 ± 0.04	<0.001
Pentadecenoic acid (c15:1n5)	0.40 ± 0.14	26.72 ± 0.30	<0.001
Palmitic acid (c16:0)	240.99 ± 25.20	223.98 ± 14.76	<0.001
Eicosapentaenoic acid (c20:5n3)	4.09 ± 0.26	7.17 ± 0.21	<0.001
Behenic acid (c22:0)	0.43 ± 0.03	28.76 ± 0.89	<0.001
Erucic acid (c22:1n9)	0.43 ± 0.10	3.35 ± 0.06	<0.001
Docosapentaenoic acid (c22:5n3)	6.41 ± 0.29	3.15 ± 1.74	<0.001
Tricosanoic acid (c23:0)	0.06 ± 0.00	19.85 ± 0.52	<0.001
Lignoceric acid (c24:0)	0.05 ± 0.00	77.22 ± 2.04	<0.001
Palmitoleic acid (c16:1n7)	20.43 ± 0.31	9.06 ± 0.30	0.001
Heptadecanoic acid (c17:0)	5.87 ± 0.72	19.85 ± 0.31	0.001
Tridecanoic acid (c13:0)	0.05 ± 0.00	0.21 ± 0.01	0.001
Nervonic acid (c24:1n9)	1.34 ± 0.07	4.85 ± 0.28	0.002
Linoleic acid (c18:2n6)	60.44 ± 1.47	80.84 ± 2.50	0.003
Octanoic acid (c8:0)	0.01 ± 0.00	0.17 ± 0.02	0.004
Decanoic acid (c10:0)	0.15 ± 0.02	1.13 ± 0.12	0.004
Linoleic acid (c18:3n6)	0.90 ± 0.05	1.43 ± 0.02	0.004
Eicosenoic acid (c20:1n9)	1.24 ± 0.24	5.59 ± 0.80	0.005
Eicosatrienoic acid (c20:3n6)	2.60 ± 0.16	7.40 ± 0.51	0.005
Arachidonic acid (c20:4n6)	31.03 ± 3.26	0.59 ± 0.10	0.006
Myristic acid (c14:0)	17.29 ± 1.70	34.18 ± 1.60	0.007
Myristoleic acid (c14:1n5)	0.89 ± 0.35	4.54 ± 0.73	0.009
Linolenic acid (c18:3n3)	4.74 ± 0.59	7.61 ± 0.02	0.011
Docosadienoic acid (c22:2n6)	0.33 ± 0.18	1.16 ± 0.03	0.011
Lauric acid (c12:0)	0.87 ± 0.08	1.79 ± 0.18	0.015
Adrenic acid (c22:4n6)	2.44 ± 0.25	1.17 ± 0.06	0.015
Oleic acid (c18:1n9)	475.35 ± 39.93	303.06 ± 22.72	0.028
Elaidic acid (c18:1tn9)	3.73 ± 0.27	4.61 ± 0.23	0.033
Heptadecenoic acid (c17:1n7)	5.26 ± 1.09	30.72 ± 10.41	0.035
Stearic acid (c18:0)	189.17 ± 12.95	148.05 ± 3.45	0.036
Undecanoic acid (c11:0)	0.01 ± 0.00	0.01 ± 0.00	0.085
Arachidic acid (c20:0)	1.00 ± 0.10	1.17 ± 0.08	0.131
Heneicosanoic acid (c21:0)	0.14 ± 0.01	0.50 ± 0.21	0.138
ΣPUFA	112.98 ± 4.62	110.52 ± 2.13	0.338
ΣSFA	457.72 ± 31.29	561.70 ± 20.37	0.059
ΣPUFA: ΣSFA	0.25 ± 0.01	0.20 ± 0.01	0.050
ΣMUFA	509.06 ± 40.10	392.51 ± 20.49	0.067
Σn-3	15.24 ± 0.20	17.93 ± 1.69	0.111
Σn-6	97.74 ± 4.45	92.60 ± 2.39	0.168
EPA	4.09 ± 0.26	7.17 ± 0.21	<0.001
TFA	1079.75 ± 76.01	1064.73 ± 42.99	0.441

Note: *p* < 0.05 and *p* < 0.01 compared with the B0 group. L* represents Lightness, a* represents Redness, b* represents Yellowness; EAA = sum of valine, lysine, phenylalanine, methionine, histidine, leucine, tryptophan, leucine, and threonine; NEAA = sum of arginine, proline, asparagine, cystine, glycine, hydroxyproline, alanine, citrulline, creatinine, taurine, glutamic acid, creatine, aminoadipic acid, cysteine, glutamine, aspartic acid, serine, tyrosine, ornithine, and choline; TAAs = total amino acids. ΣSFA: total saturated fatty acids; ΣMUFA: total monounsaturated fatty acids; ΣPUFA: total polyunsaturated fatty acids; Σn-3: total omega-3 PUFA; Σn-6: total omega-6 PUFA; EPA: eicosapentaenoic acid (C20:5n-3). Saturated fatty acids include C8:0, C10:0, C11:0, C12:0, C13:0, C14:0, C15:0, C16:0, C17:0, C18:0, C20:0, C21:0, C22:0, C23:0, C24:0. Polyunsaturated fatty acids include C18:2n6, C18:3n3, C18:3n6, C20:3n6, C20:4n6, C20:5n3, C22:2n6, C22:5n3, C22:4n6. Monounsaturated fatty acids include C14:1n5, C15:1n5, C16:1n7, C17:1n7, C18:1n9, C18:1tn9, C20:1n9, C22:1n9, C24:1n9. Total n-3 fatty acids include C18:3n3, C20:5n3, C22:5n3. Total n-6 fatty acids include C18:2n6, C18:3n6, C20:3n6, C20:4n6, C22:2n6, C22:4n6. TFA represents total fatty acid.

**Table 6:** DFMs (differential metabolites) from *longissimus Lumborum* of Tibetan sheep in the key differential enriched KEGG pathways.

Metabolic Pathway (YB VS B0)	Metabolites
Upregulation in the YB group	
Fructose and mannose metabolism	D-mannose 6-phosphate
Carbohydrate digestion and absorption	D-glucose 6-phosphate, Robison ester
Starch and sucrose metabolism	D-glucose 6-phosphate
	Robison ester
Aminoacyl–tRNA biosynthesis	Arginine, Isoleucine
	L-Lysine, DL-isoleucine
mTOR signaling pathway	Arginine, (S)-2-Amino-5-guanidinovaleric acid,
	L-Arg
Zeatin biosynthesis	Adenine, Thiomethyladenosine
	S-methyl-5′-thioadenosine
	6-Aminopurine
ABC transporters	Arginine, Isoleucine, L-Lysine
	Deoxyadenosine, L-Arg
	S-methyl-l-cysteine
	(S)-2-Amino-5-guanidinovaleric acid
	2-Amino-3-methylvaleric acid
	2,6-Diaminohexanoic acid
Phosphotransferase system (PTS)	D-glucosamine 6-phosphate
	Vitamin C, Ascorbate, Ascorbic acid
	D-mannose 6-phosphate
	D-glucosaminic acid
	D-glucose 6-phosphate
Pentose phosphate pathway (PPP)	D-glucosaminic acid
	D-Glucosaminate 2-Amino-2-deoxy-D-gluconate
Glycerophospholipid metabolism	1,2-dihexadecanoyl-sn-glycero-3-phosphocholine
	Lecithin
2-Oxocarboxylic acid metabolism	Isoleucine, N-.alpha.-acetyl-l-ornithine
	L-Lysine, 2-Amino-3-methylvaleric acid
	N-Acetylornithine, 2,6-Diaminohexanoic acid
Biosynthesis of free amino acids	Arginine, Isoleucine, L-homoserine
	N-.alpha.-acetyl-l-ornithine
	S-Adenosyl-L-homocysteine
	Lysine acid, 2,6-Diaminohexanoic acid
	2-Amino-3-methylvaleric acid
	N-Acetylornithine, L-Homoserine
	2-Amino-4-hydroxybutyric acid
Protein digestion and absorption	Arginine, Isoleucine, L-Lysine
	(S)-2-Amino-5-guanidinovaleric acid
Biofilm formation–Vibrio cholerae	D-glucose 6-phosphate, Robison ester
Biosynthesis of various secondary metabolites—part 3	Arginine, L-Lysine
	(S)-2-Amino-5-guanidinovaleric acid
Lysine biosynthesis	L-homoserine, L-Lysine
	2,6-Diaminohexanoic acid
Arginine biosynthesis	Arginine
	N-.alpha.-acetyl-l-ornithine
	(S)-2-Amino-5-guanidinovaleric acid
	N-Acetylornithine
Cysteine and methionine metabolism	L-homoserine
	S-methyl-5′-thioadenosine
	S-Adenosyl-L-homocysteine
	2-Amino-4-hydroxybutyric acid
Ascorbate and aldarate metabolism	Ascorbate, Vitamin C
HIF-1 signaling pathway	Vitamin C, Ascorbate
Vitamin digestion and absorption	Vitamin C, L-Ascorbic acid
Bile secretion	Taurochenodeoxycholate, Chenodeoxycholoyltaurine
Lipoic acid metabolism	Octanoic acid, Octanoate
Cholesterol metabolism	Taurochenodeoxycholate Chenodeoxycholoyltaurine

## Data Availability

The original contributions presented in this study are included in the article. Further inquiries can be directed to the corresponding author.

## Supplementary Materials

The following supporting information can be downloaded at https://www.mdpi.com/article/10.3390/foods14173044/s1: Figure S1: (Dataset from oats) (A) The distribution of RSD of free amino acids in QC samples of oats. (B) The distribution of RSD of fatty acids in QC samples of oats. Figure S2: (Dataset from oats) (A) The total ion chromatograms of quality control samples in positive ion modes. (B) The total ion chromatograms of quality control samples in negative ion modes. Figure S3: (Dataset from oats) Quality control of oat samples. (A) PCA analysis of all the samples based on peaks detected in positive ion modes. (B) PCA analysis of all the samples based on peaks detected in negative ion modes. Multivariate statistical analysis of oats in different regions: (C) PLS-DA and (D) OPLS-DA scores of the overall sample in the positive ion mode and permutations test of (E) PLS-DA and (F) OPLS-DA in the negative ion detection mode. Figure S4: (Dataset from sheep) Radar plot of sensory evaluation of Tibetan sheep in YB and B0 groups. Figure S5: (Dataset from sheep) (A) Total ion chromatograms of quality control samples in positive ion modes. (B) Total ion chromatograms of quality control samples in negative ion modes. Figure S6: (Dataset from sheep) Quality control of meat samples. (A) PCA analysis of all the samples based on peaks detected in positive ion modes. (B) PCA analysis of all the samples based on peaks detected in negative ion modes. Multivariate statistical analysis of oat in different regions: (C) PLS-DA and (D) OPLS-DA scores of the overall sample in the positive ion mode and permutations test of (E) PLS-DA and (F) OPLS-DA in the positive ion detection mode. Table S1: Standard curve of free amino acids and fatty acids in oat. Table S2: Standard curve of carbohydrates in oats. Table S3: Parameters of GC-MS. Table S4: Scoring criteria of sensory evaluation of meat. Table S5: Detailed results of differential metabolites in the oats in the positive and negative ion detection modes (OPLS-DA VIP > 1 and *p*-value < 0.05) (YX vs. GX). Table S6: Detailed results of differential metabolites in the longissimus lumborum in the positive and negative ion detection modes (OPLS-DA VIP > 1 and *p*-value < 0.05) (YB vs. B0). Formula (S1): Calculation of the content of free amino acids in oats. Formula (S2): Calculation of the content of fatty acids in oats. Formula (S3): Calculation of the content of carbohydrates in oats.
