# Supplementary material for: Preliminary Study on the Mechanism of the Influence of Saline Oat Pasture on Muscle Metabolism and Meat Quality of Tibetan Sheep
Source: Foods. 2025 Aug 29;14(17):3044. doi: 10.3390/foods14173044 (PMC12427911; doi:10.3390/foods14173044)
Supplement: Supplementary file 1 [file foods-14-03044-s001.zip › foods-3789392-supplementary.pdf]

Supplementary Material

S1 Supplementary Figures and Tables

S1.1 Supplementary Figures

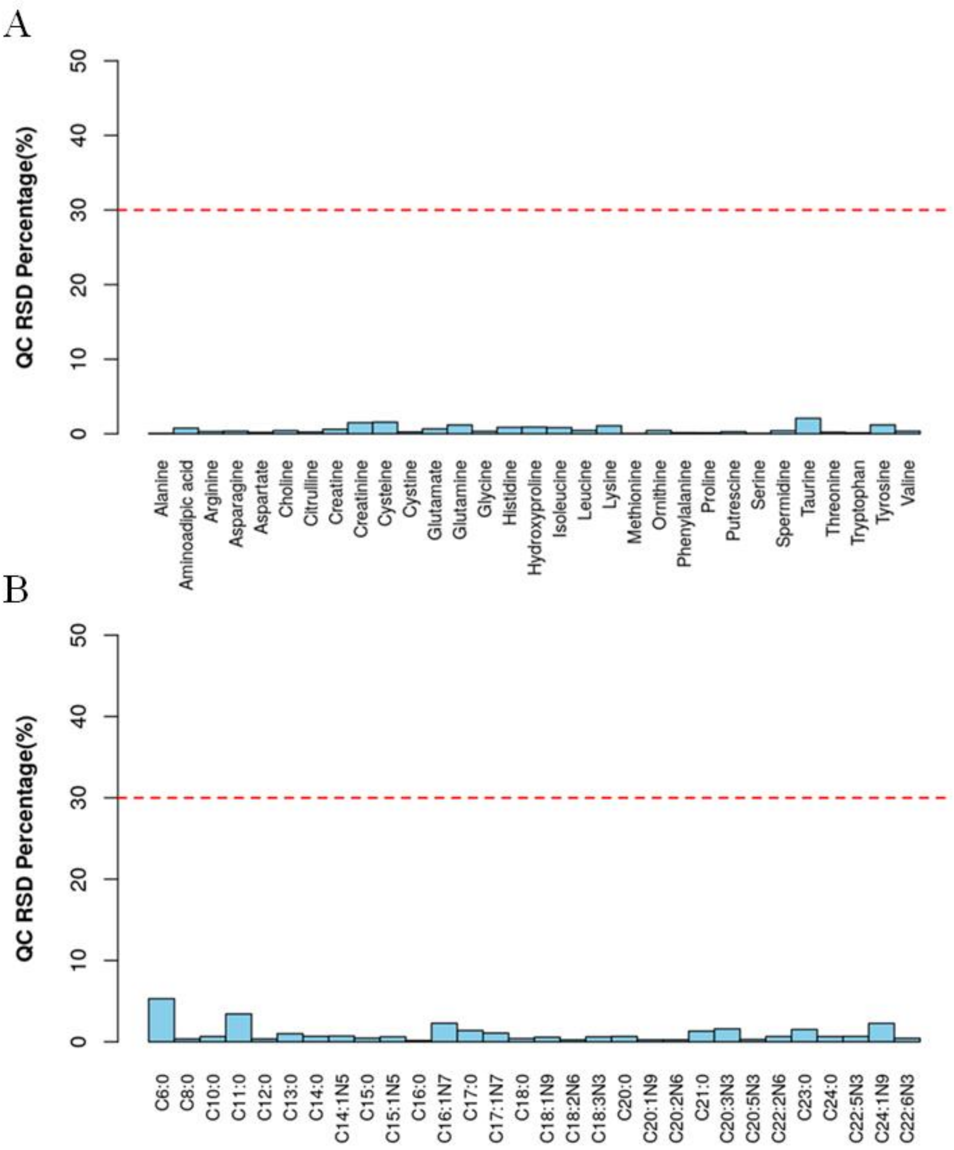

Supplementary Figure S1. (Dataset from oat) (A) The distribution of RSD of free amino acids in QC samples of oat. (B) The distribution of RSD of fatty acids in QC samples of oat.

A

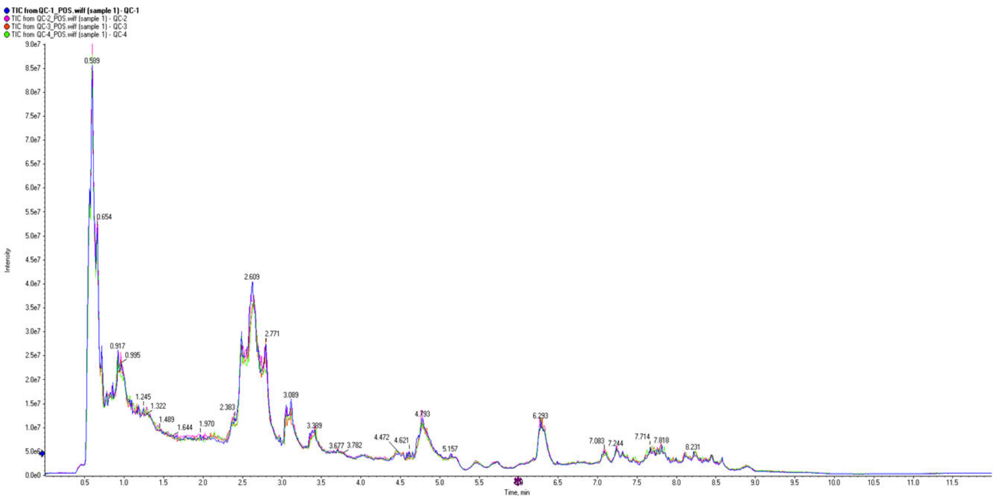

B

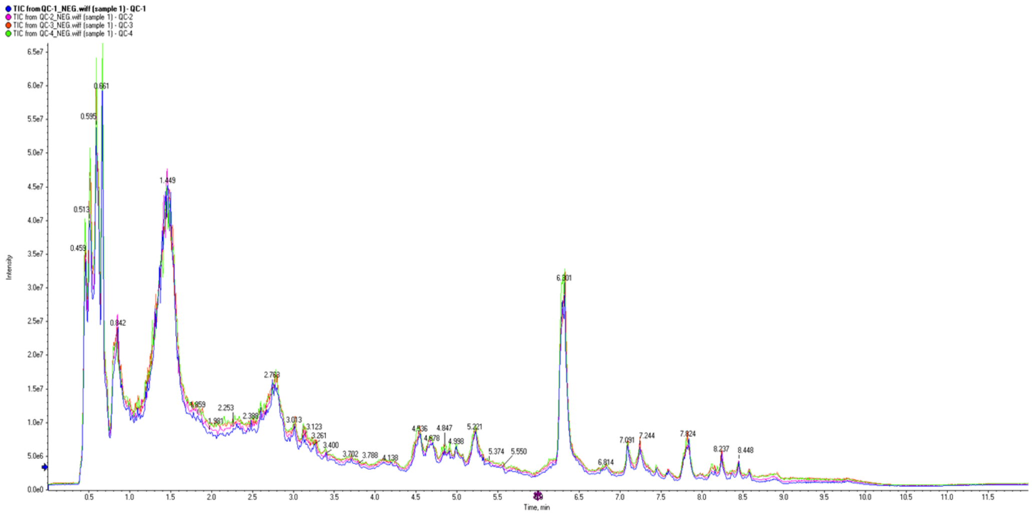

Supplementary Figure S2. (Dataset from oat) Quality control and differential metabolite analysis of oat between YX and GX groups in metabolomics. (A) The total ion chromatograms of quality control samples in positive ion modes. (B) The total ion chromatograms of quality control samples in negative ion modes.

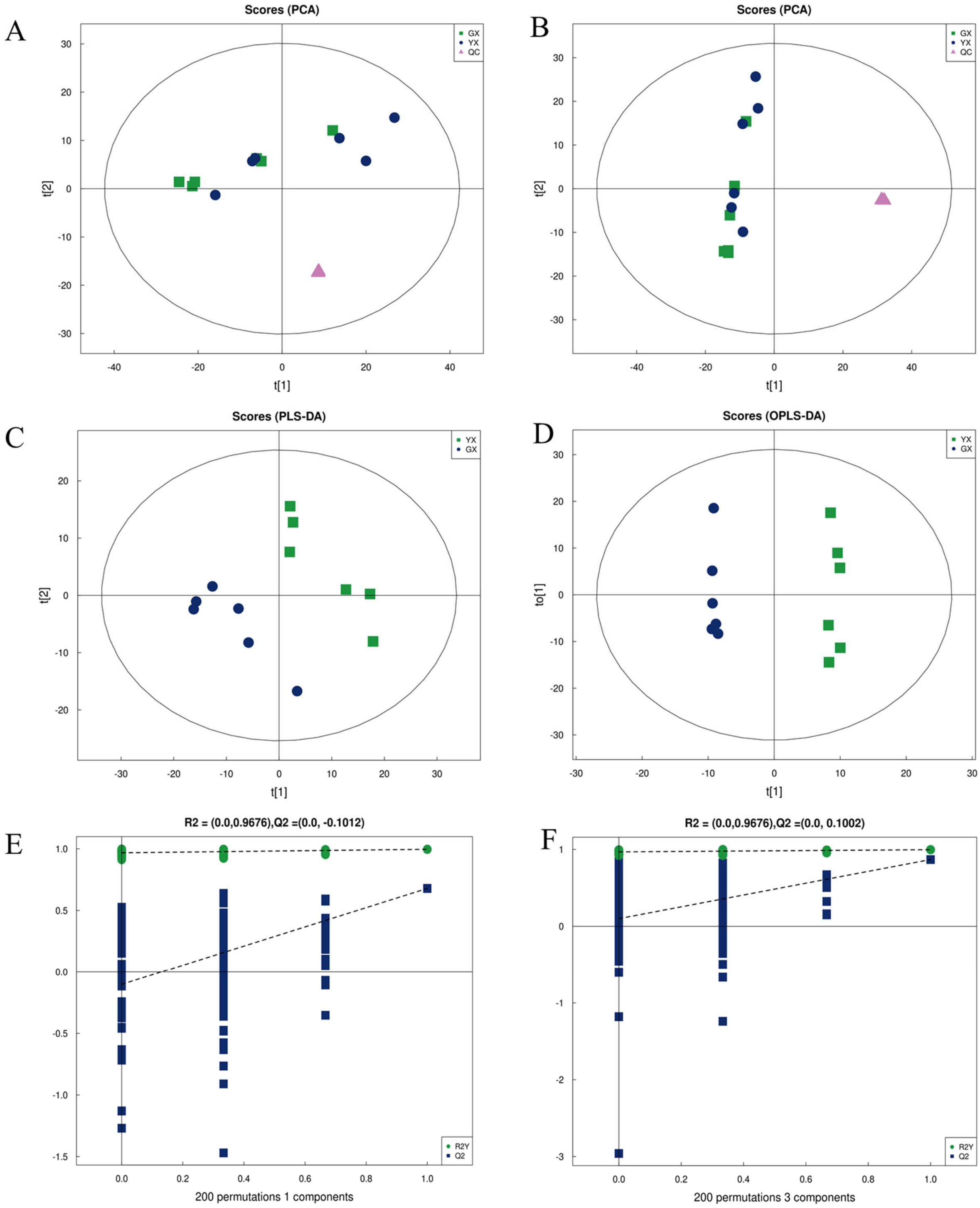

Supplementary Figure S3. (Dataset from oat) Quality control of oat samples. (A) PCA analysis of all the samples based on peaks detected in positive ion modes. (B) PCA analysis of all the samples based on peaks detected in negative ion modes. Multivariate statistical analysis of oat in different regions: (C) PLS-DA, (D) OPLS-DA scores of the overall sample in the positive ion mode and permutations test of (E) PLS-DA, (F) OPLS-DA in the negative ion detection mode.

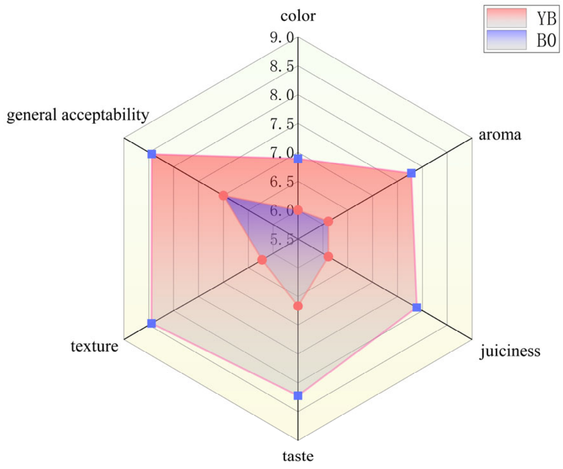

Supplementary Figure S4. (Dataset from sheep) Radar plot of sensory evaluation of Tibetan sheep in YB and B0 groups.

A

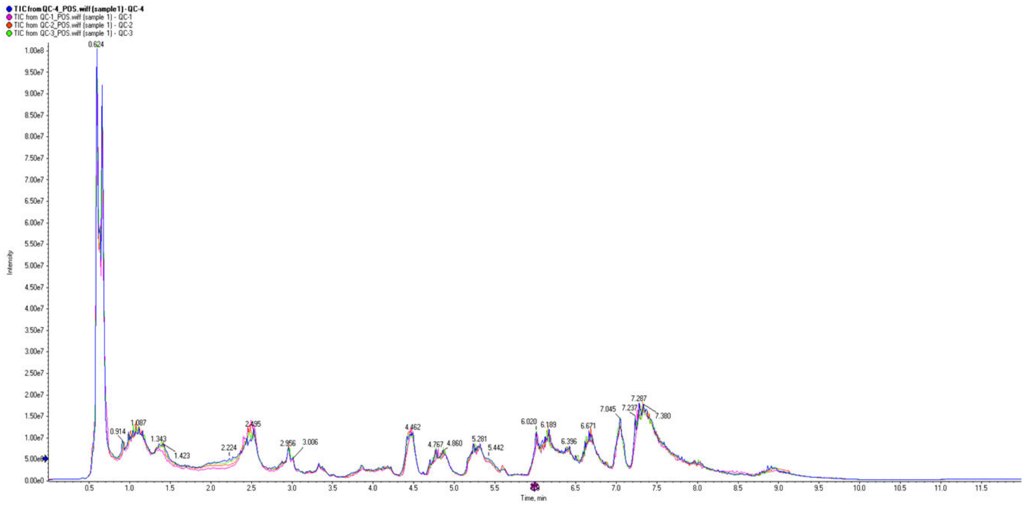

B

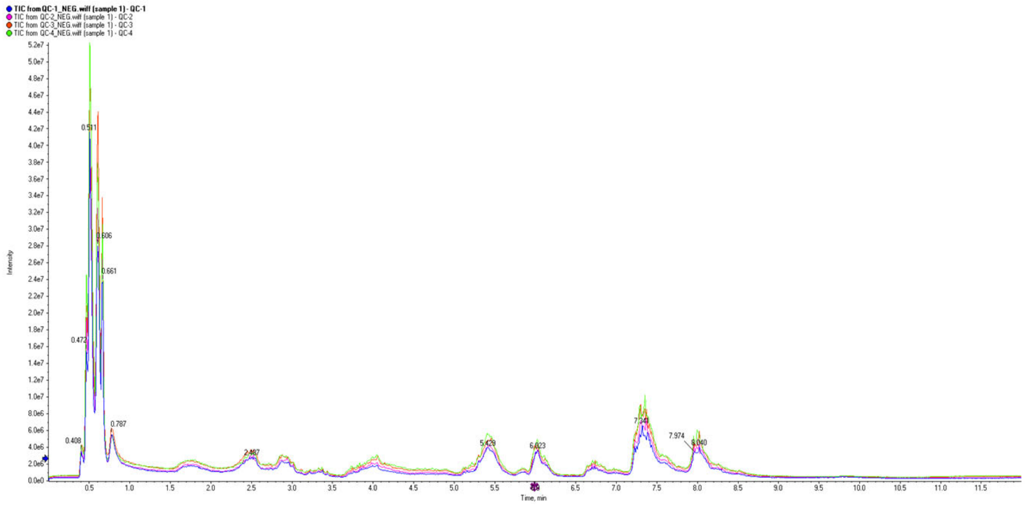

Supplementary Figure S5. (Dataset from sheep) (A) The total ion chromatograms of quality control samples in positive ion modes. (B) The total ion chromatograms of quality control samples in negative ion modes.

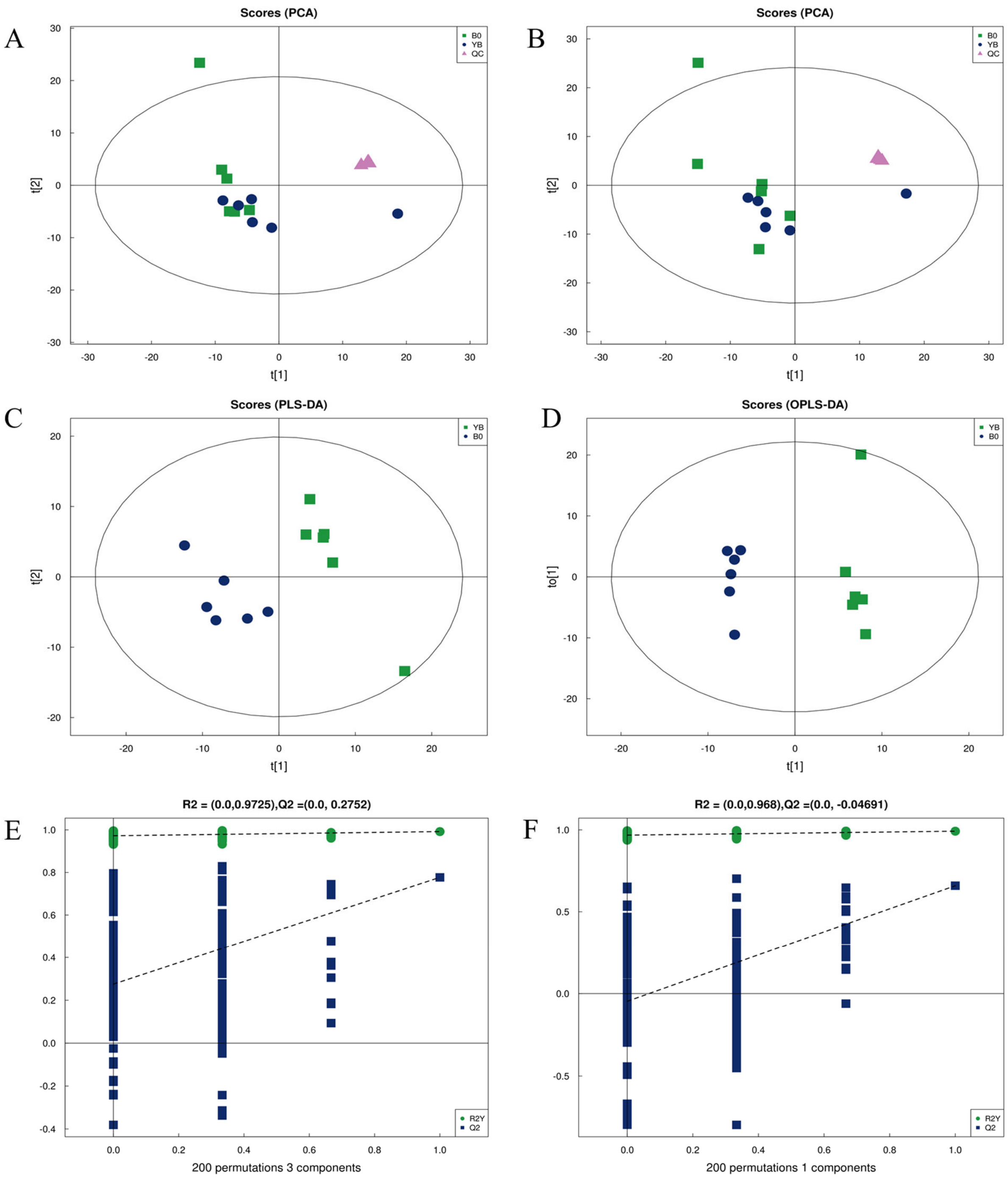

Supplementary Figure S6. (Dataset from sheep) Quality control of meat samples. (A) PCA analysis of all the samples based on peaks detected in positive ion modes. (B) PCA analysis of all the samples based on peaks detected in negative ion modes. Multivariate statistical analysis of forage in different regions: (C) PLS -DA, (D) OPLS-DA scores of the overall sample in the positive ion mode and permutations test of (E) PLS -DA, (F) OPLS-DA in the positive ion detection mode.

S1.2 Supplementary Tables

Supplementary Table S1. Standard curve of free amino acids and fatty acids in forage.

Metabolite Name	Linear	R	Linear range(umol/L)	LOD(umol/L)	LOQ(umol/L)	ULOQ (umol/L)
**Standard curve of free amino acids**						
Alanine	y = 2476.88404 x + 3.23235e4	r = 0.99923	0.1-500	0.02	0.1	500
Aminoadipic acid	y = 0.00189 x + -0.00594	r = 0.99923	0.1-500	0.02	0.1	500
Arginine	y = 1.80942e-4 x + 0.00197	r = 0.99931	0.1-500	0.02	0.1	500
Asparagine	y = 0.01799 x + -0.15421	r = 0.99805	0.1-500	0.02	0.1	500
Aspartate	y = 0.00176 x + 0.00419	r = 0.99888	0.1-500	0.02	0.1	500
Choline	y = 2.79032e-4 x + 0.01368	r = 0.99814	0.1-500	0.02	0.1	500
Citrulline	y = 0.03715 x + -0.07392	r = 0.99927	0.1-500	0.02	0.1	500
Creatine	y = 0.07538 x + -0.06340	r = 0.99850	0.1-500	0.02	0.1	500
Creatinine	y = 0.10109 x + 0.02636	r = 0.99951	0.1-500	0.02	0.1	500
Cysteine	y = 8.15866e-4 x + -0.00182	r = 0.99985	0.1-500	0.02	0.1	500
Cystine	y = 6.60987e-5 x + -8.24650e-4	r = 0.99990	0.1-500	0.02	0.1	500
Glutamate	y = 7.05822e-4 x + 0.03055	r = 0.99918	0.1-500	0.02	0.1	500
Glutamine	y = 5.58596 x + 3.96352	r = 0.99965	0.1-500	0.02	0.1	500
Glycine	y = 1.46141e-6 x + 6.27616e-5	r = 0.99837	0.1-500	0.02	0.1	500
Histidine	y = 3.55138e-4 x + -0.00841	r = 0.99754	0.1-500	0.02	0.1	500

Hydroxyproline	y = 0.03167 x + -0.03298	r = 0.99939	0.1-500	0.02	0.1	500
Isoleucine	y = 2.47631e-4 x + 0.01789	r = 0.99817	0.1-500	0.02	0.1	500
Leucine	y = 2.60337e-4 x + 0.01111	r = 0.99891	0.1-500	0.02	0.1	500
Lysine	y = 0.00919 x + 0.62060	r = 0.99874	0.1-500	0.02	0.1	500
Methionine	y = 3.95413e-5 x + 0.00365	r = 0.99931	0.1-500	0.02	0.1	500
Ornithine	y = 0.00667 x + 0.04286	r = 0.99978	0.1-500	0.02	0.1	500
Phenylalanine	y = 4.22812e-5 x + 0.00150	r = 0.99923	0.1-500	0.02	0.1	500
Proline	y = 2.80809e-4 x + 0.00444	r = 0.99858	0.1-500	0.02	0.1	500
Putrescine	y = 0.01857 x + -0.02582	r = 0.99873	0.1-500	0.02	0.1	500
Serine	y = 3.36147e-4 x + 3.31385e-4	r = 0.99957	0.1-500	0.02	0.1	500
Spermidine	y = 0.02985 x + -0.39303	r = 0.99977	0.1-500	0.02	0.1	500
Taurine	y = 4.76765e-4 x + 0.00161	r = 0.99884	0.1-500	0.02	0.1	500
Threonine	y = 3.21775e-4 x + 0.00278	r = 0.99979	0.1-500	0.02	0.1	500
Tryptophan	y = 0.00811 x + -0.02238	r = 0.99895	0.1-500	0.02	0.1	500
Tyrosine	y = 9.76536e-4 x + 0.00218	r = 0.99835	0.1-500	0.02	0.1	500
Valine	y = 2.84462e-4 x + -0.00279	r = 0.99984	0.1-500	0.02	0.1	500
**Standard curve of fatty acids**						
Butyric acid	y = 0.260576 * x +1.695842E-005	1	0.002-50	0.0004	0.002	100
Hexanoic acid	y = 0.853574 * x -1.996885E-005	1	0.002-50	0.0004	0.002	100

Octanoic acid	y = 1.228385 * x -1.030001E-004	0.9999	0.002-50	0.0004	0.002	100
Decanoic acid	y = 1.225923 * x -2.981816E-004	0.9999	0.004-100	0.0004	0.002	100
Undecanoic acid	y = 1.301301 * x -1.530991E-004	0.9998	0.002-50	0.0004	0.002	100
Lauric acid	y = 1.333405 * x +8.053487E-005	1	0.004-100	0.0004	0.002	100
Tridecanoic acid	y = 1.166860 * x +1.469826E-007	1	0.002-50	0.0004	0.002	100
Myristic acid	y = 1.405073 * x -0.002863	1	0.002-50	0.0004	0.002	100
Myristoleic acid	y = 0.480293 * x +0.004840	0.9998	0.002-50	0.0004	0.002	100
Pentadecanoic acid	y = 1.319373 * x -1.343773E-005	1	0.002-50	0.0004	0.002	100
10z-pentadecenoic acid	y = 0.489969 * x -8.810019E-004	1	0.002-50	0.0004	0.002	100
Palmitic acid	y = 1.279840 * x +0.003447	1	0.004-100	0.0004	0.002	100
Palmitoleic acid	y = 0.392884 * x +0.002342	1	0.002-50	0.0004	0.002	100
Heptadecanoic acid	y = 1.159803 * x -1.215672E-004	1	0.004-100	0.0004	0.002	100
10z-heptadecenoic acid	y = 0.385177 * x +2.633965E-005	0.9999	0.002-50	0.0004	0.002	100
Stearic acid	y = 1.248027 * x +0.003374	1	0.004-100	0.0004	0.002	100

Elaidic acid	y = 0.377446 * x +7.768454E-004	0.9999	0.002-50	0.0004	0.002	100
Oleic acid	y = 0.365349 * x +0.001075	1	0.004-100	0.0004	0.002	100
Linolelaidic acid	y = 0.492162 * x -3.826577E-005	1	0.002-50	0.0004	0.002	100
Linoleic acid	y = 0.488592 * x +1.670843E-005	1	0.002-50	0.0004	0.002	100
γ-linoleic acid	y = 0.421819 * x +9.416722E-005	1	0.004-100	0.0004	0.002	100
α-linolenicacid	y = 0.557570 * x -1.641098E-004	1	0.002-50	0.0004	0.002	100
Arachidic acid	y = 1.173948 * x -5.641273E-005	1	0.004-100	0.0004	0.002	100
11z-eicosenoic acid	y = 0.407721 * x +9.387631E-004	1	0.002-50	0.0004	0.002	100
11z,14z-eicosadienoic acid	y = 0.462416 * x +5.715936E-005	1	0.002-50	0.0004	0.002	100
8z,11z,14z-eicosatrienoic acid	y = 0.406248 * x +1.519996E-005	1	0.002-50	0.0004	0.002	100
11z,14z,17z-eicosatrienoic acid	y = 0.532455 * x +1.275701E-004	1	0.002-50	0.0004	0.002	100
Arachidonic acid	y = 0.448848 * x -3.336744E-005	0.9999	0.002-50	0.0004	0.002	100
5z,8z,11z,14z,17z-	y = 2.138585 * x -9.271162E-004	1	0.002-50	0.0004	0.002	100

eicosapentaenoic acid						
Heneicosanoic acid	y = 1.104803 * x +9.088751E-004	1	0.002-50	0.0004	0.002	100
Behenic acid	y = 0.250704 * x +3.977031E-005	1	0.004-100	0.0004	0.002	100
Erucic acid	y = 0.401425 * x +1.347127E-004	1	0.002-50	0.0004	0.002	100
13z,16z-docosadienoic acid	y = 0.428606 * x +3.431677E-005	1	0.002-50	0.0004	0.002	100
Adrenic acid	y = 0.420663 * x +1.846959E-005	0.9999	0.002-50	0.0004	0.002	100
7z,10z,13z,16z,19z-docosapentaenoic acid	y = 0.467222 * x +5.569229E-005	0.9999	0.002-50	0.0004	0.002	100
4z,7z,10z,13z,16z-docosapentaenoic acid	y = 0.413003 * x -2.659799E-005	1	0.002-50	0.0004	0.002	100
4z,7z,10z,13z,16z,19z-docosahexaenoic acid	y = 0.390827 * x +2.569570E-004	0.9999	0.002-50	0.0004	0.002	100
Tricosanoic acid	y = 1.018892 * x +2.655343E-005	1	0.002-50	0.0004	0.002	100
Lignoceric acid	y = 0.959526 * x +5.169503E-004	1	0.004-100	0.0004	0.002	100
Nervonic acid	y = 0.464333 * x +4.350010E-005	0.9999	0.002-50	0.0004	0.002	100

Supplementary Table S2. Standard curve of carbohydrates in forage.

Index	Equation	R^2	LLOQ	ULOQ
2-Deo-ribose	y = 0.234565 x - 1.397087E-004	0.99561288	0.029	5
Xylose	y = 0.499610 x - 2.327373E-004	0.993414509	0.009	5
D-Ara	y = 0.592638 x - 2.475254E-004	0.993726223	0.014	5
Ribose	y = 0.803125 x + 3.474493E-004	0.999207262	0.021	5
Xylulose	y = 0.988330 x + 0.003149	0.999299797	0.021	5
Ribono-1-4-lactone	y = 0.160414 x - 6.114101E-005	0.998577295	0.1	5
Xylitol	y = 1.145413 x - 4.472466E-004	0.999061598	0.01	5
Lev	y = 0.217581 x - 1.244303E-004	0.997977834	0.021	5
Rha	y = 0.427569 x - 2.713368E-004	0.998345782	0.048	5
Arabinitol	y = 1.099802 x + 1.803210E-004	0.998967158	0.04	5
Fuc	y = 0.048389 x - 2.461162E-005	0.994575458	0.025	5
Deo	y = 0.141567 x - 6.743920E-005	0.993438324	0.032	5
1-5-Anh	y = 0.410255 x - 2.161059E-004	0.992832928	0.027	5
Met	y = 0.401484 x - 1.573916E-004	0.992241692	0.022	5
Fru	y = 0.615515 x - 0.004406	0.992294272	0.006	50
Man	y = 0.276248 x - 1.913223E-004	0.993540251	0.008	5
Gal	y = 0.172883 x - 1.059422E-004	0.996935026	0.011	5

Glu	y = 0.285443 x - 0.001347	0.993518973	0.011	50
Sorbitol	y = 1.547737 x - 1.749951E-004	0.996378269	0.007	50
Glucuronic-A	y = 0.253019 x - 1.244356E-004	0.99165536	0.009	5
Gal-A	y = 0.172867 x - 1.000721E-004	0.991901091	0.012	5
2-Ace-2-Deo-D-Glucosamine	y = 0.039881 x - 2.256294E-005	0.992968674	0.028	5
Inositol	y = 0.564638 x - 2.891097E-004	0.993869797	0.013	25
Ribose-5-pho-Ba	y = 0.010744 x - 5.583250E-006	0.997662532	0.074	5
Man-6-pho	y = 0.004469 x - 3.326033E-006	0.995864502	0.023	5
Phe	y = 0.347461 x - 2.013665E-004	0.990860677	0.004	5
Suc	y = 1.007351 x - 0.015490	0.990059522	0.007	50
Lac	y = 0.040655 x - 1.714121E-005	0.996900666	0.019	5
Cel	y = 0.125571 x - 5.584382E-005	0.993622648	0.015	25
Mal	y = 0.122375 x - 6.726263E-005	0.995287615	0.029	25
Tre	y = 0.681649 x - 4.009184E-004	0.990866023	0.003	25
Raffinose	y = 0.054376 x - 5.141744E-005	0.990470637	0.021	25

Supplementary Table S3. The parameters of GC-MS.

**Mass spectrometry conditions**	**Parameter**
Injection volume	1 μL
split mode	5:1

Carrier Gas	Helium
Column	DB-5MS (30 m x 0.25 mm x 0.25 μm)
Column Flow	1 mL/min
Oven Temperature Ramp	Held on 1 min at 160°C, raised to 200°C at a rate of 6°C/min, raised to 270°C at a rate of 10°C/min, raised to 320°C at a rate of 20°C/min and kept for 5.5 min.
Transfer Line Temperature	
Ion Source Temperature	230°C
Quad Temperature	150°C
Electron Energy	70 eV

Supplementary Table S4. The scoring criteria of sensory evaluation of meat.

**Evaluation projects**	**Score/Description**		
	**1-4**	**5-7**	**8-10**
color	meat color is dark, without luste	dark meat, the luster is weak	the meat color is light red, shiny
aroma	no meat aroma	more meaty aroma	full of meat aroma
juiciness	there is little juice, and it is obviously dry when chewed	it has a certain amount of juice, and there is no obvious dryness when chewing	the meat juice is rich when chewing, and the mouth is always moist
taste	the meat is coarse and difficult to chew.	the meat is tender and delicious, but the chewiness is average	fresh and delicious, with good chewiness

texture	The organization is poor and the meat is old and hard.	Organizational status is average, elasticity is average	The organization state is uniform and close, the elasticity is good, and the internal structure is uniform
general acceptability	difficult to accept	more acceptable	easily accepted.

Supplementary Table S5. The detailed results of differential metabolites in the forage in the positive and negative ion detection mode (OPLS-DA VIP > 1 and *P value < 0.05*) (YX *vs* GX).

**Name**	**Adduct**	**m/z**	**Rt(s)**	**VIP**	***P***	**FC**	**Variation**
**Metabolites in the positive ion**							
Arachidonoylthiophosphorylcholine	[M+H]+	784.55	149.01	2.61	< 0.001	3.52	↑
5-methyl-2'-deoxycytidine	[M+H-$C_5H_8O_3$]+	126.06	209.67	2.86	< 0.001	7.27	↑
Thymine	[M+H]+	127.05	105.94	2.53	< 0.001	6.41	↑
Deoxyadenosine	[M+H]+	252.11	146.97	13.50	< 0.001	8.75	↑
Cytosine	[M+H]+	112.05	259.12	3.30	< 0.001	2.39	↑
L-pyroglutamic acid	[M+H]+	130.05	410.31	6.56	< 0.001	0.52	↓
Puberanidine	[M+H]+	543.31	157.54	1.93	< 0.001	0.42	↓
Trigonelline	[M+H]+	138.05	309.67	7.70	< 0.001	3.23	↑
Lavandulol	(M+H-H_2O)+	137.13	156.13	1.52	< 0.001	3.25	↑
2'-o-methyladenosine	[M+H]+	282.12	110.48	1.46	0.01	2.98	↑

4-Aminobutyric acid(GABA)	[M+H]+	104.07	388.22	3.18	0.01	0.75	↓
D-turanose	[M+NH4]+	360.15	377.62	10.96	0.01	0.58	↓
Lactulose	[M+H-H_2O]+	325.11	377.64	8.97	0.01	0.61	↓
4-hydroxybutanoic acid lactone	[M+H]+	87.04	388.29	3.25	0.01	0.73	↓
Glu-Val-Arg	[M+H]+	403.23	440.55	1.05	0.01	0.49	↓
Melezitose	[M+NH_4]+	522.20	434.77	9.15	0.01	0.49	↓
Ile-Ala	[M+H]+	203.14	255.26	1.10	0.01	2.15	↑
D-psicose	[M+H-2H_2O]+	145.05	377.66	3.37	0.01	0.62	↓
Tetrahydrocorticosterone	[M+H]+	351.25	38.65	1.08	0.01	1.93	↑
Cytidine	[2M+H]+	487.17	259.12	3.59	0.02	5.45	↑
2'-deoxycytidine	[2M+H]+	455.19	220.53	2.98	0.02	43.82	↑
Dl-ethionine	[M+H]+	164.09	370.85	1.16	0.02	0.42	↓
Oleic acid methyl ester	[M+H-CH_6O_2]+	247.24	36.67	1.35	0.02	0.84	↓
Leucylleucine	[M+H]+	245.18	198.62	2.35	0.02	2.53	↑
N-tris(hydroxymethyl)methylglycine	[M+H]+	180.07	370.50	1.42	0.03	31.97	↑
DL-glutamine	[M+H]+	147.07	409.35	3.69	0.03	0.60	↓

1-Aminocyclopropanecarboxylic acid	(M+H-H_2O)+	84.04	410.46	2.32	0.03	0.60	↓
4.alpha.-mannobiose	[2M+Na]+	707.22	377.62	1.60	0.04	0.69	↓
4-hydroxyatorvastatin lactone	[M+H]+	557.25	266.45	1.17	0.04	0.24	↓
2-Hydroxyadenine	(M+H)+	152.05	248.25	2.11	0.04	1.86	↑
1-o-b-d-glucopyranosyl sinapate	[M+H]+	387.14	42.68	1.96	0.05	0.13	↓
**Metabolites in the negative ion**							
Trehalose	[M-H]-	341.11	407.30	8.14	< 0.001	0.47	↓
Palatinose	[2M-H]-	683.22	404.76	2.43	< 0.001	0.28	↓
Succinate	[M-H]-	117.02	411.07	1.60	< 0.001	0.34	↓
Blood group b trisaccharide	[M-H]-	487.17	405.45	2.70	< 0.001	0.24	↓
3-hydroxy-7,8,2',3'-tetramethoxyflavone	[M-H]-	357.08	58.80	2.99	< 0.001	0.50	↓
Ile-Pro	[M-H]-	227.07	119.88	1.38	< 0.001	5.72	↑
His-ser	[M-H]-	241.08	105.49	5.71	< 0.001	8.00	↑
5'-deoxyadenosine	[M-H]-	250.09	151.08	1.11	< 0.001	9.02	↑
Gentiopicroside	[M+FA-H]-	401.13	377.72	8.29	0.01	0.59	↓
D-Galactarate	(M-H_2O-H)-	191.02	302.83	3.08	0.01	0.57	↓
Sucrose	[M-H]-	341.11	377.74	19.05	0.01	0.61	↓

Marticin	[M-H]-	375.07	107.18	1.37	0.01	6.28	↑
(e)-n-[2-hydroxy-2-(4-hydroxyphenyl)ethyl]-3-(4-hydroxy-3-methoxyphenyl)prop-2-enamide	[M-H]-	328.12	56.77	1.13	0.01	4.88	↑
Palmitic acid	[M-H]-	255.23	49.97	7.83	0.01	2.20	↑
Deoxyguanosine	[M-H]-	266.09	249.22	1.28	0.01	7.52	↑
Raffinose	[M-H]-	503.16	433.67	8.84	0.01	0.34	↓
D-arabinose	[M-H-2H_2O]-	113.02	279.66	1.93	0.02	0.68	↓
Erythritol	(M+Na-2H)-	143.03	280.14	1.42	0.02	0.71	↓
4-hydroxyphenethyl alcohol	[M-H]-	137.06	159.99	1.05	0.03	0.08	↓
8-[4,5-dihydroxy-6-(hydroxymethyl)-3-[3,4,5-trihydroxy-6-(hydroxymethyl)oxan-2-yl]oxyoxan-2-yl]-5,7-dihydroxy-2-(4-hydroxyphenyl)chromen-4-one	[M-H]-	593.15	315.55	1.21	0.03	1.93	↑
L-Arabinono-1,4-lactone	[M-H]-	147.03	116.08	2.39	0.04	0.59	↓
Isoorientin	[M-H]-	447.09	388.54	1.80	0.04	0.48	↓
9-oxo-10e,12z,15z-octadecatrienoic acid	[M-H]-	291.19	40.48	1.17	0.04	1.87	↑
His-Lys	[M-H]-	282.08	281.69	1.34	0.04	2.30	↑

5,7-dihydroxy-2-(4-hydroxyphenyl)-6-[3,4,5-trihydroxy-6-(hydroxymethyl)oxan-2-yl]-8-(3,4,5-trihydroxyoxan-2-yl)chromen-4-one	[M-H]-	563.14	349.42	4.36	0.05	2.47	↑
Melibiose	[M-H]-	341.11	446.48	1.20	0.05	0.65	↓
D-Allose	(2M-H)-	359.12	318.87	2.35	0.05	0.63	↓

Supplementary Table S6. The detailed results of differential metabolites in the longissimus lumborum in the positive and negative ion detection mode (OPLS-DA VIP > 1 and *P value < 0.05*) (YB *vs* B0).

Name	Adduct	m/z	Rt(s)	VIP	*P*	FC	Variation
**Metabolites in the positive ion**							
D-glucosaminic acid	[M+H-H_2O]+	178.08	294.89	4.29	< 0.001	5.11	↑
L-palmitoylcarnitine	[M+H]+	400.34	180.24	11.75	< 0.001	0.30	↓
6-dimethylamino-4-ketohexanoic acid	[M+H]+	174.11	349.36	4.41	< 0.001	0.16	↓
Leucylleucine	[M+H]+	245.18	197.41	1.68	< 0.001	2.00	↑
L-homoserine	[M+H-H_2O]+	102.05	55.25	1.14	< 0.001	1.89	↑
3-hydroxybutyrylcarnitine	[M+H]+	248.15	334.89	19.72	< 0.001	2.13	↑

Lithocholylglycine	[M+Na]+	456.33	270.01	1.06	< 0.001	0.21	↓
4-nitroanisole	[M+H]+	154.03	252.74	1.39	< 0.001	0.42	↓
Deoxyadenosine	[M+H-$C_5H_8O_3$]+	136.06	109.34	2.89	0.01	1.77	↑
S-methyl-l-cysteine	[M+H]+	136.02	254.97	1.07	0.01	0.35	↓
(r)-butyrylcarnitine	[M+H]+	232.16	267.12	30.57	0.01	2.24	↑
Lauroyl-l-carnitine	[M+H]+	344.28	189.96	3.68	0.01	0.34	↓
1,2-dihexadecanoyl-sn-glycero-3-phosphocholine	[M+Na]+	756.55	67.96	1.04	0.01	1.36	↑
4-benzofuranethanamine, 2,3-dihydro-.alpha.-methyl-	[M+H]+	178.13	199.68	1.09	0.01	2.24	↑
6-[3-[(3,4-dimethoxyphenyl)methyl]-4-methoxy-2-(methoxymethyl)butyl]-4-methoxy-1,3-benzodioxole	[M-H_2O+H]+	415.21	39.40	6.85	0.01	1.19	↑
Lpc 18:2	[M+H]+	520.34	193.80	1.52	0.01	1.47	↑
1-palmitoyl-2-docosahexaenoyl-sn-glycero-3-phosphocholine	[M+H]+	806.56	42.57	1.62	0.02	1.43	↑
Lpc 18:1	[M+H]+	522.35	191.97	3.38	0.02	1.53	↑
1h-1,2,4-triazol-3-amine	[M+H]+	85.03	266.51	1.11	0.02	1.88	↑

(2r)-3-hydroxyisovaleroylcarnitine	[M+H]+	262.16	308.41	5.82	0.02	2.39	↑
3-oxazolidinecarboxylic acid, 2,2-dimethyl-4-(1-oxo-2-hexadecyn-1-yl)-, 1,1-dimethylethyl ester, (4s)-	[M+Na]+	458.34	262.32	1.06	0.02	0.28	↓
S-methyl-5'-thioadenosine	[M+H]+	298.10	110.01	9.97	0.02	1.86	↑
Isobutyryl-l-carnitine	[2M+H]+	463.30	266.73	2.76	0.02	10.72	↑
Oleoyl-l-carnitine	[M+H]+	426.36	176.83	11.53	0.03	0.51	↓
Dl-threonine methyl ester	[M+H]+	134.08	74.26	3.12	0.03	2.54	↑
Arachidonoylthiophosphorylcholine	[M+H]+	784.58	94.28	2.31	0.03	1.78	↑
Decanoyl-l-carnitine	[M+H]+	316.25	197.50	2.37	0.03	0.36	↓
Creatinine	[M+H]+	114.07	180.92	6.46	0.04	1.19	↑
1-Stearoyl-sn-glycerol 3-phosphocholine(LPC(18:0))	[M+H]+	524.37	161.22	1.58	0.04	1.78	↑
S-Adenosyl-L-homocysteine	(M+H)+	385.13	496.71	1.34	0.04	1.87	↑
Tomatidin	[M+H]+	416.34	202.55	4.39	0.04	0.59	↓
L-Lysine	(2M+Na)+	315.20	513.14	1.19	0.04	2.32	↑
D-glucose 6-phosphate	[M+H-H_2O]+	243.02	491.26	2.38	0.05	3.59	↑

4-hydroxy-l-isoleucine	[M+H-CH_2O_2]+	102.09	372.01	1.85	0.05	0.73	↓
DL-isoleucine	[M+H]+	132.10	302.12	1.72	0.05	1.53	↑
**Metabolites in the negative ion**							
N-acetyl-l-methionine	[M-H]-	190.05	202.17	1.20	< 0.001	2.07	↑
Leu-Glu	[M-H]-	259.13	380.64	1.13	< 0.001	1.76	↑
Physcion	[M-H]-	283.05	173.02	1.31	< 0.001	1.67	↑
Val-Glu	[M-H]-	245.11	402.15	1.33	< 0.001	2.11	↑
Adenine	[M-H]-	134.05	104.00	9.40	< 0.001	2.93	↑
Benzoic acid, 2-[[(2z)-3-(3,4-dimethoxyphenyl)-1-oxo-2-propen-1-yl]amino]-3-hydroxy-	[M-H]-	342.08	105.40	1.22	< 0.001	2.15	↑
Nis(monooleoylglycero) phosphate (s,r isomer)	[M-H]-	773.55	40.57	4.50	< 0.001	0.43	↓
(.+/-.)-11-nor-.delta.9-tetrahydrocannabinol-9-carboxylic acid	[2M-H]-	687.37	31.00	1.11	< 0.001	0.79	↓
Hippuric acid	[M-H]-	178.05	194.55	3.09	< 0.001	2.47	↑
3-dehydrocholic acid	[M-H]-	405.26	193.85	1.17	< 0.001	0.23	↓
Allantoin	[M-H]-	157.04	185.60	2.29	< 0.001	1.72	↑
Leu-Phe	[M-H]-	277.15	176.87	1.22	< 0.001	1.72	↑
Val-Phe	[M-H]-	263.14	189.54	1.22	0.01	1.84	↑
Arginine	[M-H]-	173.09	302.69	1.02	0.01	2.11	↑

3'-O-methyladenosine	(M+K-2H)-	318.06	103.21	1.42	0.01	2.57	↑
Pc (18:1e/20-hdohe)	[M+Hac-H]-	892.60	140.82	1.08	0.01	1.65	↑
Uridine 5'-monophosphate	[M-H]-	323.03	470.52	2.06	0.02	1.51	↑
D-glucosamine 6-phosphate	[M-H]-	258.04	493.18	1.02	0.02	2.76	↑
Isoleucine	[M-H]-	130.09	292.97	4.55	0.02	1.71	↑
2,6-di-tert-butylphenol	[M-H]-	205.16	34.02	2.09	0.02	1.43	↑
Octanoic acid	[M-H]-	143.11	44.63	1.07	0.02	1.71	↑
Val-Ile	[M-H]-	229.16	204.83	2.20	0.02	2.32	↑
Labetalol	[M-H]-	327.16	37.29	1.37	0.03	0.58	↓
Vitamin c	[M-H-$C_2H_4O_2$]-	114.98	325.05	1.51	0.03	1.43	↑
Ethyl p-coumarate	[M-H]-	191.07	109.87	1.74	0.03	2.99	↑
Indolelactic acid	[M-H-CH_2O_2]-	158.06	213.95	1.75	0.03	1.98	↑
Pc (16:1e/19,20-epdpe)	[M+Hac-H]-	864.57	41.47	1.00	0.03	1.87	↑
1-stearoyl-2-arachidonoyl-sn-glycero-3-phospho-(1'-sn-glycerol)	[M-H]-	797.55	40.44	2.22	0.04	0.63	↓
N-.alpha.-acetyl-l-ornithine	[M-H]-	173.09	389.27	1.78	0.04	1.38	↑

Eplerenone hydroxy acid	[M-H-$C_2H_6O_4$]-	337.20	28.50	5.90	0.04	0.56	↓
D-mannose 6-phosphate	[M-H]-	259.02	493.21	12.63	0.04	4.74	↑
Cinchonine	[M-H]-	293.18	28.64	8.46	0.04	0.66	↓
1-oleoyl-sn-glycero-3-phosphoethanolamine	[M-H]-	478.29	198.12	1.76	0.04	1.40	↑
Val-Asp	[M-H]-	231.10	408.40	1.44	0.04	2.56	↑
Taurochenodeoxycholate	[M-H]-	498.29	156.35	1.51	0.04	2.85	↑
1,5-dicaffeoylquinic acid	[M-H-$C_{18}H_{12}O_6$]-	191.06	384.27	1.42	0.04	0.55	↓
Ala-Tyr	[M-H]-	251.10	298.45	1.39	0.04	2.44	↑
Pc (16:1e/8,9-epete)	[M+Hac-H]-	838.56	143.32	1.26	0.05	1.48	↑

S1.3 Supplementary Formulas

Free amino acid content (umol/g) of forage, calculated according to formula S1.

$$The\ Content\ of\ free\ amino\ acid\ (umol/g) = C \times \frac{V}{m} \times n \qquad \text{(S1)}$$

The meanings of the letters in the formula: C is the software-calculated concentration of component x in the sample; V is the corresponding volume of the corresponding calibration point; m is the weighing amount of the original sample; and n is the multiple of dilutions.

Fatty acid content (umol/g) of forage, calculated according to formula S2.

$$The\ Content\ of\ fatty\ acid\ (umol/g) = C \times \frac{V}{m} \times n \qquad \text{(S2)}$$

The meanings of the letters in the formula: C is the software-calculated concentration of component x in the sample; V is the corresponding volume of the corresponding calibration point; m is the weighing amount of the original sample; and n is the multiple of dilutions.

The Content of carbohydrates(mg/g) in forage, calculated according to formula S3.

$$The\ Content\ of\ Carbohydrates\ (mg/g) = c \times \frac{V_1 \times V_2}{V_3 \times m \times 1000000} \quad \text{(S3)}$$

The meanings of the letters in the formula: C is the concentration value (μg/mL) obtained by substituting the integrated peak area ratio in the sample into the standard curve; V_1 is the volume of the solution used for volume determination (μL); V_2 is the volume of the sample extract added during the sample extraction process (μL); V_3 is the volume of supernatant collected during the sample extraction process (μL); and m is the weighed sample mass (g).
